# The impact of elevated temperature and salinity on microbial communities and food selectivity in heterotrophic nanoflagellates in the Boye River

**DOI:** 10.1093/ismeco/ycaf049

**Published:** 2025-03-21

**Authors:** Lisa Boden, Dana Bludau, Guido Sieber, Aman Deep, Daria Baikova, Gwendoline M David, Una Hadžiomerović, Tom L Stach, Jens Boenigk

**Affiliations:** Department Biodiversity, University of Duisburg–Essen, Essen, NRW 45141, Germany; Department Biodiversity, University of Duisburg–Essen, Essen, NRW 45141, Germany; Center for Water and Environmental Research, University of Duisburg–Essen, Essen, NRW 45141, Germany; Department of Engineering and Natural Sciences, Westphalian University of Applied Sciences, Recklinghausen, NRW 45665, Germany; Department Biodiversity, University of Duisburg–Essen, Essen, NRW 45141, Germany; Center for Water and Environmental Research, University of Duisburg–Essen, Essen, NRW 45141, Germany; Department Biodiversity, University of Duisburg–Essen, Essen, NRW 45141, Germany; Department of Engineering and Natural Sciences, Westphalian University of Applied Sciences, Recklinghausen, NRW 45665, Germany; Department Environmental Microbiology and Biotechnology, University of Duisburg–Essen, Essen, NRW 45141, Germany; Department of Plankton and Microbial Ecology, Leibniz Institute of Freshwater Ecology and Inland Fisheries (IGB), Stechlin, BB 12587, Germany; Center for Water and Environmental Research, University of Duisburg–Essen, Essen, NRW 45141, Germany; Department Environmental Microbiology and Biotechnology, University of Duisburg–Essen, Essen, NRW 45141, Germany; Center for Water and Environmental Research, University of Duisburg–Essen, Essen, NRW 45141, Germany; Environmental Metagenomics, Research Center One Health Ruhr, University Alliance Ruhr, University of Duisburg–Essen, Essen, NRW 45141, Germany; Department Biodiversity, University of Duisburg–Essen, Essen, NRW 45141, Germany; Center for Water and Environmental Research, University of Duisburg–Essen, Essen, NRW 45141, Germany

**Keywords:** food web, predator–prey interactions, microbial communities, freshwater ecology, bacterivory, heat stress, salinization, CARD-FISH, microscopy, amplicon sequencing

## Abstract

Microbial predator–prey interactions play a crucial role in aquatic food webs. Bacterivorous protists not only regulate the quantity and biomass of bacterial populations but also profoundly influence the structure of bacterial communities. Consequently, alterations in both the quantity and quality of protist bacterivory can influence the overall structure of aquatic food webs. While it is well-documented that changes in environmental conditions or the occurrence of abiotic stressors can lead to shifts in microbial community compositions, the impact of such disturbances on food selection remains unknown. Here, we investigated the effects of elevated temperature and salinization on food selectivity of heterotrophic nanoflagellates by monitoring the uptake of preselected target bacteria via catalyzed reporter deposition fluorescence *in situ* hybridization and fluorescence microscopy. Our results indicate that salinization, but not increased temperature, significantly increased the flagellates’ selection against *Microbacterium lacusdiani* (Actinomycetota). However, the effect of the reduced grazing pressure was counterbalanced by the negative effect of increased salinity on the growth of Actinomycetota. Our results suggest that the effect of stressors on the feeding behavior of protistan predators may strongly affect the composition of their prey community, when bacterial taxa are concerned that are less sensitive to the particular stressor.

## Introduction

Microbial processes and predator–prey interactions form the basis of food webs in aquatic environments and stabilize their ecosystem functions [1]. Shifts in feeding and food preferences of bacterivorous protists may alter composition and functioning of prokaryotic communities [2, 3]. Understanding the response of the protist’s food selection to environmental changes is therefore crucial for understanding and predicting ecosystem functioning when exposed to stress.

Climate change and anthropogenic activities are affecting environmental conditions in freshwater ecosystems by raising water temperatures, causing decreased oxygen levels and intensified thermal stratification in freshwater bodies [4, 5]. In addition to the gradual increase in global water temperatures, ecosystems are increasingly subjected to more frequent and intense heatwaves, during which water temperatures are projected to rise by 4–6°C [5, 6]. Furthermore, factors like agriculture, urbanization, and mining activities are increasing salinity in lakes and rivers worldwide [7]. Chloride (Cl^−^) concentrations in natural rivers typically range from 1 to 20 mg/l, but pollution can rise them up to 200 mg/l [8]. Even higher concentrations, reaching 3 g/l in mining effluent [9] and 8 g/l in freshwater bodies affected by winter road salting [8], have also been observed. Rising temperatures, salinization, and their combination pose serious threats, particularly to freshwater ecosystems [10]. Significant shifts in freshwater microbial communities due to heat stress [11–14], as well as salinization [15–18], have been well documented. Such shifts in prokaryotic and microeukaryotic communities can significantly impact microbial predator–prey interactions by altering composition of both predator and prey communities. Additionally, elevated temperatures typically reduce cell size and increase growth rates of bacteria, primary producers and predatory protists [19–22], which can alter microbial biomass and further impact aquatic food webs. However, it remains unknown whether abiotic disturbances also affect the active food selection of grazing protists, and to what extent stressor-induced changes in their feeding behavior contribute to observed shifts in prey populations.

An essential component in aquatic food webs is the group of heterotrophic nanoflagellates (HNFs). While HNFs are an important food source for macroinvertebrates, they are also among the main consumers of bacteria, controlling the abundance and biomass of their prey and significantly shaping the taxonomic and morphological composition of prokaryotic communities [23, 24]. For example, the presence of HNFs typically shifts prey populations toward smaller, fast-growing bacteria by preferentially targeting larger bacterial cells [25, 26]. Additionally, HNFs play a vital role in the remineralization of organic matter and nutrients, thereby stimulating bacterial growth and viral proliferation in aquatic habitats [27–29].

The flagellates’ food selection can be influenced by prey characteristics such as cell size, motility, physicochemical surface properties, or nutritional quality [30, 31], as well as available food concentrations and the filling statutes of the predators’ food vacuole [32, 33]. Inducible defense mechanisms by the prey and feedback interactions between predator and prey can further affect the feeding behavior of HNFs [34, 35]. While these biotic influences on HNF food selection are well-documented, the impact of abiotic environmental factors on HNF’s feeding behavior remains less well understood. Previous studies have demonstrated that elevated temperatures can significantly increase the grazing pressure of nanoflagellates on bacteria [36, 37]. However, it remains unknown whether heat stress also impacts the active food selection of the predators. Regarding salinization, little is known about its impact on microbial predator–prey interactions. While studies have shown that increased salinity can significantly alter prokaryotic community composition [38, 39], thereby affecting prey availability, the direct effects of salinization on the feeding behavior or food selection of bacterivorous protists in freshwater habitats have not, to our knowledge, been investigated.

To directly investigate the impact of warming and salinization, both individually and in combination, on predator–prey interactions, we examined the effects of a 5°C temperature increase and a 2.5 g.l^−1^ increase in sodium chloride concentration on microbial community composition and the food selection of HNFs using three identical mesocosm experiments. These experiments were conducted using water and sediment collected from the Boye River in the Ruhr area of Germany during the fall of 2022 and 2023. We expect that stressor-induced shifts will occur in both prokaryotic and microeukaryotic communities, with more pronounced effects on prokaryotes due to their closer link to geochemical and physical factors [40]. Building on Staniczenko *et al.* [41], who proposed that predators adapt to environmental changes by incorporating new prey species into their diets, we hypothesize that HNF food selection will decrease under stress but recover once the stressors are removed. Understanding potential stressor-induced changes in HNF’s feeding behavior could help explain observed shifts in bacterial prey populations, as alterations in food selection are likely to influence prokaryotic community composition. By investigating both individual and combined exposure to these stressors, we aim to gain a better understanding of how multiple stressors interact. We expect that simultaneous exposure will have more pronounced effects on microbial communities and predator–prey interactions compared to exposure to a single stressor, due to the high frequency of additive effects of multiple stressors in freshwater habitats [42]. The temporal separation of our replicates captures natural variation in freshwater habitats, likely resulting in differences in starting communities. Despite these initial differences, we expect the stressors to have similar effects on community composition and to cause consistent changes in HNF food selection across replicates, allowing for more generalizable conclusions.

## Materials and methods

### Experimental setup and stressor experiment

The AquaFlow systems (Fig. S1), situated in a greenhouse of the University Duisburg-Essen and previously described by Graupner *et al.* [43], were used to investigate the effects of predicted heatwaves [6] and mining effluent intrusion [9] on freshwater microbial communities. In total three experiments with equal set-ups were run in September 2022, October 2022 and September 2023. Briefly, four identical circular mesocosms, each consisting of three steel tanks (40 l, 40 l, 270 l) were connected by two steel flow channels (5 cm width, 4 m length; 10 cm width, 2 m length), a pump (Eheim compactON 1000, Deizisau, Germany) and an aquarium chiller (TR/TC20, TECO SRL, Ravenna, Italy) to mimic riffle and pool sequences of natural rivers (Fig. S1). Each mesocosm was filled with approximately 40 l of natural sediment and 365 l of water from the Boye catchment (51°33′19.7″N 6°56′38.3″E). In a fifth system, hereafter referred to as “donor system”, the broad flow channel was removed to include a larger tank (storage tank 1, Fig. S1), which allowed for more water to circulate in this system (~1600 l). Before being added to the flow channels, nine parts of sediment were homogenized and mixed with one part of fine particulate organic matter (FPOM) to increase the nutrient content in the systems, supporting a more naturalistic riverine environment. To simulate natural leaf fall and its contribution to the riverine food web, leaves (*Alnus glutinosa*), previously collected from trees and air-dried, were cut into small pieces and packed into mesh bags (3 × 5 cm^2^; 0.5 cm mesh 0.6–0.8 g leaves/bag). The leaf bags were pre-incubated with water and leaf litter from the Boye stream for 5 days to promote colonization by a microbial community representative of that in the target stream. After pre-incubation, 35 leaf bags were fixed on top of the sediment in each narrow channel using stainless steel thread to prevent them from being transported downstream and accumulating in the tanks. Two larger mesh bags filled with leaf litter from the Boye were placed in the smaller tanks of each mesocosm for 3 days to further promote microbial colonization of the leaves and establish a more robust community of microbial decomposers. The water was prefiltered with a 100 μm mesh to exclude larger debris and invertebrates. The flow velocity was adjusted to 12 cm.s^−1^ in the narrow channel and 6 cm.s^−1^ in the broader channel. Natural daylight served as a light source. All mesocosms were acclimatized for 10 days at 15°C. During the acclimatization phase all systems were connected once a day to exchange water among the systems and homogenize the microbial communities among the mesocosms. On day one, referring to the first day after the acclimatization phase, the abiotic conditions remained the same in all systems. On day two, mesocosms were exposed to the respective stressors, i.e. water temperature was increased from 15°C to 20°C in the temperature and combination treatments and 2.5 g.l^−1^ sodium chloride were added to the salt and combination treatments (Fig. S1). Abiotic conditions remained unchanged in the control treatment and the donor system.

On day six, three liters of water were collected from each mesocosm and split into 250 ml dialysis flasks (Slide-A-LyzerTM dialysis flask, 20 K MWCO, 250 ml, Thermo ScientificTM, Waltham, Massachusetts, USA). The remaining water from the control, temperature, salt and combination treatments was drained and all four mesocosms were refilled with water from the donor system. The dialysis flasks were then placed into the big tank (tank 1, Fig. S1) of each corresponding mesocosm, and the water temperature was maintained at 15°C in all systems until the end of the experiment.

### Sampling and grazing experiment

Water samples were collected daily, excluding days seven and nine. On day two, samples were taken 3 h after stressor onset, and on day six, 3 h after stressor removal. Each sampling day, 250 ml of water were collected from each system and filtered onto white polycarbonate filters (diameter 47 mm, pore size 0.2 μm, Millipore GTTP 04700, Eschborn, Germany). The filters were air-dried, frozen in liquid nitrogen, and stored at −80°C until further processing for deoxyribonucleic acid (DNA) extraction. Furthermore, grazing experiments were performed on each sampling day, each conducted in three replicates. For each experiment, 100 ml of water were collected from each of the four treatments. Two bacterial strains, *Limnohabitans* spp. strain IID5 (Pseudomonadota) and *Microbacterium lacusdiani* DSM 29188 (Actinomycetota), were added to each water sample (final concentration (fc.) 10^6^ cell.ml^−1^ for each bacterium). *Limnohabitans* and *Microbacterium* were chosen as common and widespread representatives [44, 45] of two of the most abundant bacterial phyla in freshwater communities, Pseudomonadota and Actinomycetota, respectively [46–48]. The bacteria had previously been cultured in NSY medium (Nutrient broth, Peptone from soybean (Bacto Soytone), Yeast extract) [49], centrifuged at 2830 g at room temperature (RT) for 10 min to remove the medium and resuspended in sterile filtered water from the Boye. For each sampling day, freshly cultured bacteria were transferred from NSY medium and resuspended into a new aliquot of the sterile filtered water, ensuring that only recently transferred bacteria (less than 24 h) were used in the experiments. Additionally, 0.3 μl fluorescently labeled beads (Fluoresbrite® YG Microspheres, Calibration Grade 0.50 μm, Polysciences, Inc. Warrington, Pennsylvania, USA) were added to the water samples (fc. 10^6^ beads.ml^−1^). A 15 ml aliquot was collected immediately after addition of bacteria and beads and fixed by adding Lugol’s solution (fc. 1%) and paraformaldehyde (fc. 2%) as described in Piwosz *et al.* [50]. A second aliquot was fixed 12 min after addition. The subsamples were decolorized by adding 3% sodium thiosulphate solution [50] and kept at 4°C overnight for complete sample fixation. The samples were then filtered onto white polycarbonate filters (diameter 25 mm, pore size 0.2 μm, Millipore GTTP 02500, Eschborn, Germany), air-dried and stored at −20°C until further processing.

### Deoxyribonucleic acid extraction and deoxyribonucleic acid amplicon sequencing

To extract DNA from the filtered water samples, each filter was carefully fragmented into small pieces using sterile tweezers and mixed with 2 mm Zirconia Beads and 0.3 mm Garnet Beads. Then, 100 μl Proteinase K, 5 μl RNase A and 900 μl TNES (for buffer see [51]) were added, followed by bead-beating for 20 min at 2400 rpm and incubation for 20 min at 56°C and 1400 rpm. DNA was isolated from 300 μl aliquots of the lysate (2 replicates per sample) using spin column extraction with a vacuum manifold as described in Buchner [52]. The extracted DNA was cleaned up with carboxylated-magnetic beads and PEG-NaCl buffer by following Buchner [53] using 40 μl of DNA and 80 μl of cleanup solution. The V4 region of the 16S ribosomal ribonucleic acid (rRNA) gene and the V9 region of the 18S rRNA gene were amplified via a two-step polymerase chain reaction (PCR) approach using the Multiplex PCR Plus Kit (Quiagen, Hilden, Germany) with 1 μl DNA and the primers 515f/806r [54] for the 16S rRNA gene and 1389F/1510R [55] for the 18S rRNA gene. The PCR product was cleaned up as described by Buchner [53] and 2 μl of the DNA were used for the second PCR to add individual tags to each sample. PCR reagents and cycling conditions of both PCRs are listed in Table S1. The final PCR product was cleaned up and normalized using magnetic beads [52]. The samples were pooled and the resulting libraries concentrated via spin column cleanup protocol [52], before being sent for paired-end sequencing on Illumina NovaSeq (16S: 2 × 250 bp, CeGat, Tübingen, Germany; 18S: 2 × 150, Azenta Life Sciences, Leipzig Germany).

### Amplicon sequence analysis

Raw reads were processed using the Natrix2 amplicon workflow (v1.0.0) [56]. Briefly, the pipeline incorporated steps for primer removal, paired-end assembly with Pandaseq (v2.11) [57] and filtering the reads using an alignment threshold score of 0.9 and minimum and maximum sequence length of 100 and 600 bp, respectively. Further steps for dereplication (100% sequence similarity) using cd-hit (v4.8.1) [58] and removing chimeric sequences by VSEARCH (v2.15.2) [59] were included. AmpliconDuo (v1.1) [60] was used to discard artificial sequences based on a split sample approach, before the remaining sequences were clustered into operational taxonomic units (OTUs) using the default parameters of Swarm (v3.0.0) [61]. For taxonomic classification, mothur (v1.47.0) [62] was used to align the 16S rRNA gene sequences against the Silva database (v138.1) [63] and the 18S rRNA gene sequences against the PR^2^ database (v4.14.0) [64] using a minimum confidence value of 80. MUMU (v0.0.1) [65] was used for post-clustering and removed artificial OTUs. Custom Python scripts (Table S2) were used to subtract the maximum read counts of OTUs found in the negative controls from the corresponding read counts in each sample. The read abundances of PCR replicates were combined before filtering out OTUs that were unique to a single sample or had fewer than 100 reads. OTUs classified as Metazoa or Embryophyta were removed to exclude larger animals and land plants from the dataset. Additionally, Archaea were removed to focus on bacteria, which serve as the main food source for HNFs. Hereafter, “prokaryotic community” and “bacterial community” will be used interchangeably.

Statistical and graphical analysis were conducted in RStudio (v4.3.3) [66]. The 1 - Simpson index, hereafter referred to as Simpson’s Diversity index, was visualized using the phyloseq package (v1.46.0) [67]. Normalized and log-transformed data (DESeq2, design: ~replicate+treatment, v1.42.0) [68] were ordinated using a principal coordinates analysis (PCoA) based on Bray-Curtis distances and visualized with phyloseq and ggplot2 (v3.5.1) [69]. Taxonomic compositions were analysed with the R packages fantaxtic (v0.2.1) [70], microViz (v0.12.1) [71] and ggplot2. To assess the treatment effects on microbial community composition, a distance-based Redundancy Analysis (db-RDA) was performed based on Bray–Curtis dissimilarity using vegan (v2.6.8) [72] and dplyr (v1.1.4) [73]. Treatment was defined as explanatory variable. To account for the inherent impact of the experimental setup, hereafter referred to as “temporal effect”, and the variation among replicates, timepoint and replicate were included as random effects. The first db-RDA axis scores were statistically compared among treatments using Kruskal–Wallis test followed by Dunn’s test using dunn.test (v1.3.6) [74]. To assess treatment effects in each replicate, separate nested permutational multivariate analyses of variance (PERMANOVAs) were conducted, accounting for time point variation as a fixed effect, based on Bray–Curtis dissimilarity matrices and using 999 permutations.

### Catalyzed reporter deposition-fluorescence *in situ* hybridization and food selectivity

All incubations, unless stated otherwise, were performed at RT. Sections of the filters prepared during the grazing experiments were cut out and embedded in 0.015% agarose by immersing them into a preheated agarose solution, placing them face-up onto a clean glass plate and letting them air-dry. The filters were removed from the glass plate by soaking them with 100% ethanol and air-dried. Endogenous peroxidases were inactivated by incubating the filters in 3% H₂O₂ for 10 min, followed by 5 min in 1 × phosphate-buffered saline (PBS, pH 7.6) and rinsing in sterile water for 1 min, before dehydrating in 100% ethanol for 30 s and air-drying. Samples were incubated in fresh lysozyme solution (10 mg.ml^−1^ in 0.1 M Tris/HCl, pH 8; 0.05 M EDTA, pH 8) for 3 min at 37°C, then washed for 5 min in sterile water. Afterward, 7 μl of HRP (horseradish peroxidase)-labeled probes (50 ng.μl^−1^ DNA, Biomeres.net GmbH, Ulm, Germany) were mixed with 2 ml hybridization buffer [75], before the filters were added and incubated overnight at 46°C. Detailed information on the probes R-BT065 [76] and HGC69a [77] is provided in Table S3. The filters were washed for 30 min at 48°C in pre-warmed washing buffer [75] and incubated in 1 × PBS (pH 7.6) for 45 min. For signal amplification 20 μl of H_2_O_2_ (0.15% v/v in 1 × PSB, pH 7.6) and 5 μl of fluorescently labeled tyramide working solution (iFluor^TM^ 647 Tyramide, AAT Bioquest, Sunnyvale, California, USA) were mixed with 2 ml amplification buffer [75]. The filters were added and incubated for 1 h at 46°C, then incubated for 10 min in 1 × PBS (pH 7.6), rinsed in sterile water for 1 min, dehydrated in 100% ethanol for 30 s and air-dried. The filters were counterstained by incubating for 10 min in a 4,6–diamidino–2–phenylindole (DAPI) solution (0.1 mg.ml^−1^), washed in sterile water for 1 min and dehydrated with 100% ethanol for 30 s. After air-drying, filters were mounted in the non-hardening and anti-bleaching mounting medium CitiFluor™ AF2 (Citifluor, Ltd., London, United Kingdom).

Samples were imaged with a BZ-X810 fluorescence microscope (Keyence, Osaka, Japan) using a 100x objective and appropriate fluorescence filters for DAPI, FITC, and Cy5 signals, along with BZ-X800 viewer software and BZ-H4XD advanced observation module. Z-stacks were acquired by capturing images at multiple focal planes with a 0.1 μm slice interval for all three channels. Images were analysed in Fiji [78], where each single-channel stack was converted to a maximum intensity projection (MIP) and merged into a multi-channel MIP.

Total counts of flagellates, bacteria, and beads per image, along with the number of bacteria and beads ingested by flagellates, were determined using the spot colocalization plugin [79]. Accuracy of these automated counts was ensured through manual inspection of 3D visualizations of a representative subset of original image stacks generated using the Volume Viewer in Fiji [78]. HNFs were identified based on size (2–20 μm) and the absence of autofluorescence in the Cy5 channel [80], ensuring the exclusion of phototrophic and mixotrophic protists as well as larger heterotrophic protists. Food selectivity was calculated using Chesson’s α-index [81] from the total abundances (A) of *Linmohabitans spp*. (L), *Microbacterium lacusdiani* (M), and beads (B), along with their ingestions (I)., i.e.: 


$$ {\mathrm{\alpha}}_{\mathrm{L}}=\frac{I_L/{A}_L}{I_L/{A}_L+{I}_M/{A}_M+{I}_B/{A}_B} $$

Chesson’s α-indices of 0.33 indicate unselective feeding, while values above 0.33 show positive selection and values below 0.33 indicate negative selection. Chesson’s α-indices among treatments were statistically compared using Kruskal–Wallis test followed by Dunn’s test with the packages readxl (v1.4.3) [82], dplyr, purrr (v1.0.2) [83] and dunn.test.

### Use of artificial intelligence

ChatGPT was used to improve grammar and readability of the manuscript. Following the use of this tool, the authors revised the text and take full responsibility for the content of this publication.

## Results

### Salinization affects food preferences of heterotrophic nanoflagellates

Cell counts for HNFs, both bacterial strains, and fluorescently labeled beads for each grazing experiment, along with the number of bacteria and beads ingested by the nanoflagellates, are summarized in Table S4.

**Figure 1:**
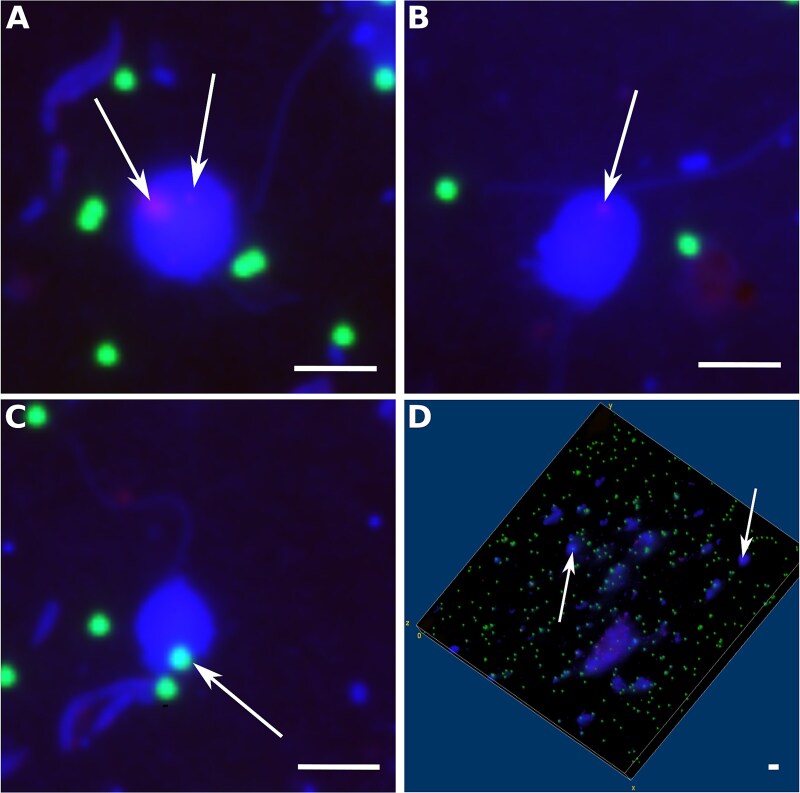
Fluorescence microscopy images showing HNFs containing different particles in their food vacuoles. Fluorescently labeled beads were added to the samples before fixation. *Limnohabitans* and *Microbacterium* were detected via CARD-FISH using HRP- labeled probes and fluorescently labeled tyramides in fixed samples. After fixation and CARD-FISH staining, all cells were counterstained with DAPI. (A) HNF with two ingested *Limnohabitans spp.* cells (arrows). (B) HNF containing an ingested *Microbacterium lacusdiani cell* (arrow). (C) HNF with an ingested fluorescently labeled bead (arrow). (D) 3D visualization of a Z-stack, highlighting the spatial distribution of ingested particles (arrows). Scale bars represent 2 μm.

All three potential food sources were ingested by the flagellates (Fig. 1). During the stressor phase, HNFs’ food selection differed significantly among the treatments for *Microbacterium* (Kruskal–Wallis test; *P* < .001), but not for the other two food sources (Kruskal–Wallis test; *Limnohabitans*: *P* = .068; beads: *P* = .068). No significant differences between the mesocosms were observed prior to addition of the stressors (Kruskal–Wallis test; *Limnohabitans*: *P* = .794; *Microbacterium*: *P* = .531; beads: *P* = .897) or during the recovery phase (Kruskal–Wallis test; *Limnohabitans*: *P* = .712; *Microbacterium*: *P* = .920; beads: *P* = .578).

**Figure 2:**
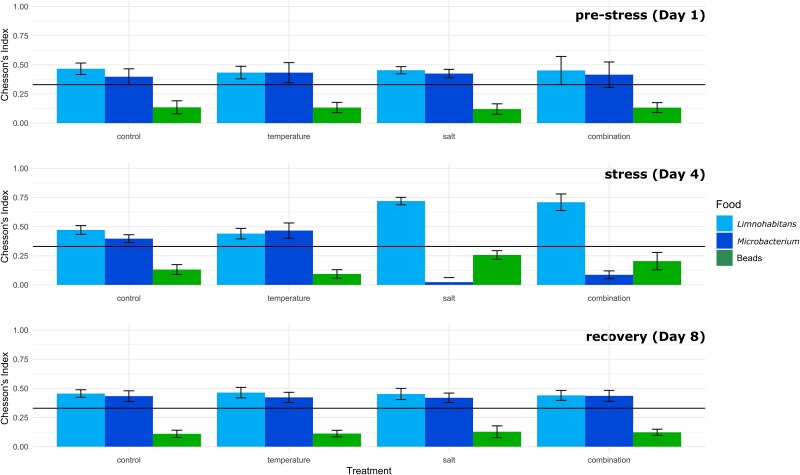
Food selectivity of heterotrophic nanoflagellates. Bar plots depicting Chesson’s alpha index for *Limnohabitans* spp*.*, *Microbacterium lacusdiani* and fluorescently labeled beads before stressor-onset, during the stressor phase and during the recovery phase. Black line indicates neutral selection. Error bars represent standard deviations.

Before stressor onset, both bacterial strains were positively selected by the flagellates, while the fluorescently labeled beads were negatively selected in all systems (Fig. 2). After the addition of the stressors, no significant differences in food selection were observed between the control and temperature treatment (Dunn’s test; *Limnohabitans*: *P* = .258; *Microbacterium: P* = .067; beads: *P* = .141), with food selection remaining positive for both bacteria and negative for the beads (Fig. 2). When exposed to increased salinity, however, the flagellates’ preference for *Microbacterium* decreased significantly (Kruskal–Wallis test; *P* = .001), resulting in strong selection against this food source (Fig. 2). Consequently, food selection for *Microbacterium* in the salt treatment differed significantly from that in the control (Dunn’s test; *P* < .001) and in the temperature treatment (Dunn’s test; *P* < .001). When simultaneously exposed to both temperature and salt treatments, hereafter referred to as combination treatment, food selection for *Microbacterium* also differed significantly compared to both control (Dunn’s test; *P* = .02) and temperature treatment (Dunn’s test; *P* < .001). No significant differences were observed between salt and combination treatment (Dunn’s test; *P* = .073), as the combination treatment also increased selection against *Microbacterium* (Fig. 2).

During the recovery phase, no significant differences were observed among the treatments (Kruskal–Wallis test; *Limnohabitans*: *P* = .712; *Microbacterium*: *P* = .920; beads: *P* = .578). After stressor removal, *Limnohabitans* and *Microbacterium* were positively selected by the flagellates, while the fluorescently labeled beads were negatively selected in all systems (Fig. 2). No significant differences in food selection were observed when comparing before stressor addition to the recovery phase for any of the food sources (Kruskal–Wallis test; *Limnohabitans*: *P* = 1.000; *Microbacterium*: *P* = .473; beads: *P* = .339).

### Salinization significantly alters composition of prokaryotic and microeukaryotic communities

Read and OTU counts for both 16S and 18S rRNA gene data are summarized in Table S5, with complete OTU tables provided as supplementary files. Analysis of the complete bacterial and microeukaryotic communities revealed a decrease in the Simpson’s Diversity Index for both prokaryotes and microeukaryotes throughout the experiment, indicating reduced overall biodiversity and shifts in community composition (Fig. S2).

**Figure 3:**
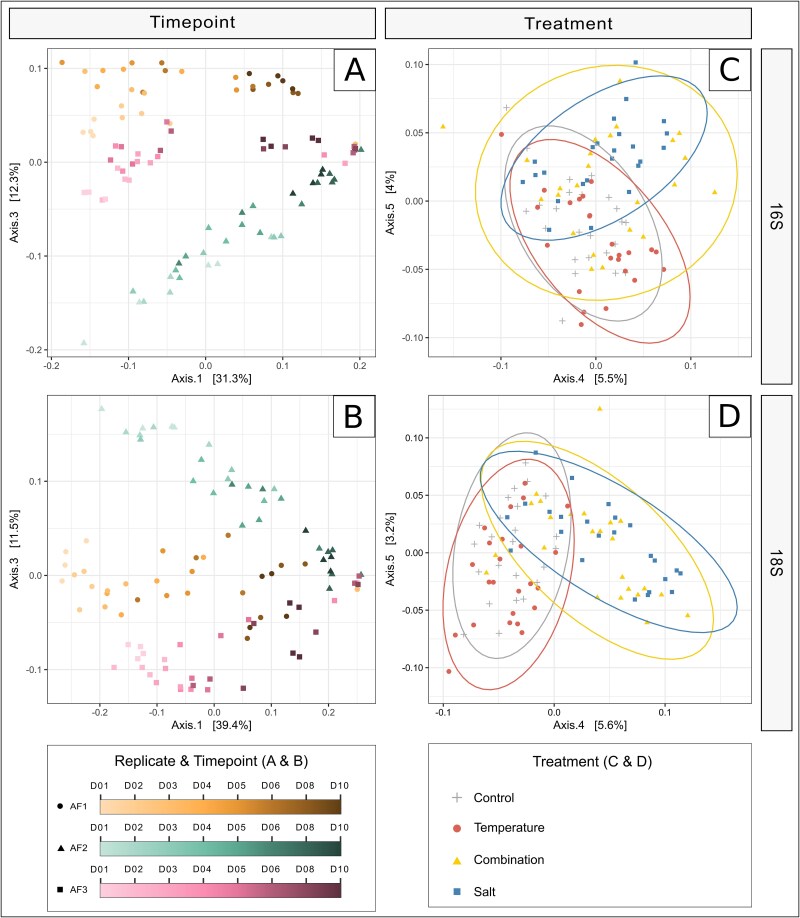
PCoA of community composition based on Bray–Curtis dissimilarity measures in water samples from all three replicates. The PCoA plots depict the natural variation between the three replicates on axis 3 and the temporal effect on the community composition on axis 1 (A and B) as well as stressor induced-effects on the axes 4 and 5 (C and D) for both the prokaryotic (A and C) and the microeukaryotic community (B and D).

PCoAs revealed a strong temporal effect on both prokaryotic (Fig. 3A) and microeukaryotic community (Fig. 3B), as well as a clear separation between the three replicates, especially at the beginning of each experiment. No treatment effects were apparent on the primary axes (Fig. S3).

Treatment effects were observed on the subsequent axes, for both bacteria (Fig. 3C) and microeukaryotes (Fig. 3D). Increased salinity significantly altered both prokaryotic (Dunn’s test; salt: *P* < .001, combination: *P* = .001) and microeukaryotic communities (Dunn’s test; salt: *P* < .001, combination: *P* < .001), while no significant differences were found between salt and combination treatment (Dunn’s test; 16S: *P* = .279; 18S: *P* = .494). Community compositions in the temperature treatment did not significantly differ from the control for bacteria (Dunn’s test; *P* = .146) or microeukaryotes (Dunn’s test; *P* = .376).

**Figure 4:**
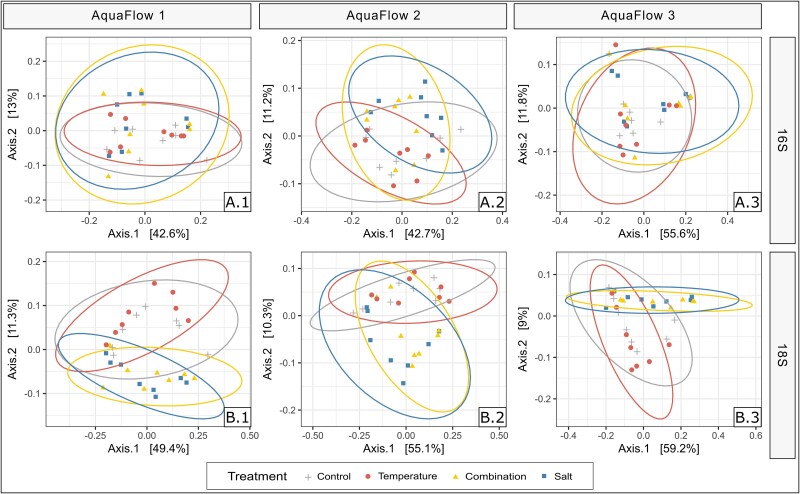
PCoA of community composition in water samples based on Bray–Curtis dissimilarity measures from the individual replicates. The PCoA plots depict stressor-induced effects on community composition of the prokaryotic (A.1–3) and microeukaryotic community (B.1–3).

Similar effects were observed in each replicate when the experiments were analysed individually. PCoAs revealed a strong temporal effect shaping both prokaryotic and microeukaryotic communities (Fig. S4). Additionally, varying treatment effects on the prokaryotic and microeukaryotic community (Fig. 4) were observed. Salinization significantly impacted the community composition of both bacteria (PERMANOVA; *P* = .001 for each experiment) and microeukaryotes (PERMANOVA; *P* = .001 for each experiment).

Effect size (R^2^), calculated from the db-RDA analysis, indicated that the salt treatment explained approximately 4.19% of the variance in the bacterial community composition, compared to 6.57% for the microeukaryotic community.

### Taxonomic composition in prokaryotic and microeukaryotic communities

The taxonomic composition of the microeukaryotic community is shown in Fig. S5. Following stressor addition, the relative abundance of Ochrophyta increased in all mesocosms, but the composition of its subgroups varied among treatments. During the stressor phase, unclassified Ochrophyta increased in the control and temperature treatments, whereas Chrysophyceae became the dominant group within Ochrophyta in the salt and combination treatments. Additionally, Choanoflagellida increased notably in the salt and combination treatments, while remaining stable in the control and temperature treatments. This shift coincided with a decline in Centroheliozoa in the salt and combination treatments. During the recovery phase, Chrysophyceae also increased in the control and temperature treatments, ultimately dominating the microeukaryotic community in all mesocosms by the end of the experiment.

The taxonomic composition of the bacterial community is shown in Fig. S6. Pseudomonadota and Bacteroidota dominated in all mesocosms throughout the experiments. Actinomycetota was the third most abundant phylum in all systems prior to stressor addition. During the stressor phase, Actinomycetota remained third most abundant phylum in the control and temperature treatment, while Cyanobacteria took this position in the salt and combination treatments. Cyanobacteria remained third most abundant phylum in both salt and combination treatments even after stressor removal.

**Figure 5:**
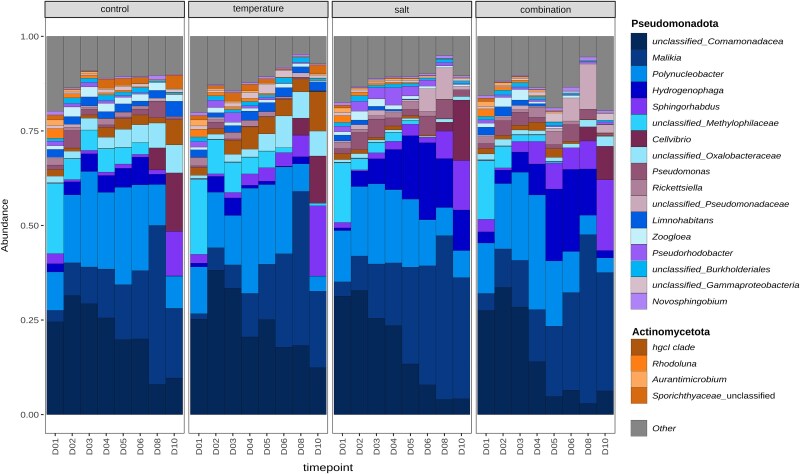
Relative abundance of Actinomycetota and Pseudomonadota. Taxonomic bar plots representing the relative abundance of the most abundant genera belonging to Actinomycetota and Pseudomonadota across the different treatments during the experiments. “Other” encompasses all additional genera of Actinomycetota and Pseudomonadota present in the samples.

Genera within Pseudomonadota and Actinomycetota exhibited varying responses to changing environmental conditions (Fig. 5). While Pseudomonadota remained the dominant group in all systems, the relative abundance of *Limnohabitans* increased during the stressor phase in the control (+0.1%) and temperature treatments (+0.4%) but decreased in the salt (−0.9%) and combination treatments (−0.6%). Whereas the relative abundance of Microbacteriaceae, closely related to *Microbacterium*, decreased in all systems, though the extent of this decrease varied by treatment (control: −2.0%; temperature: −1.2%; salt: −0.7%; combination: −1%).

## Discussion

Microbial food webs are critical for maintaining the stability of aquatic ecosystem functions [2, 3]. Understanding how abiotic disturbances affect microbial predator–prey interactions is therefore essential for predicting the impacts of climate change and anthropogenic activities on aquatic life.

Our results indicate that increased salinity affected the microbial community and significantly altered HNFs’ food selection. In contrast, no significant changes in community composition or food selection were observed in the temperature treatment. Thus, the combined exposure to salinization and heat stress caused effects similar to the salt treatment alone. This was unexpected as previous studies demonstrated that temperature variations can significantly alter microbial communities [11, 13, 84]. However, those studies examined prolonged heat stress or seasonal variations, while our study focused on short-term temperature increases, suggesting that microbial communities may be more resistant to short-term heat stress.

This study focused on the feeding behavior of HNFs, while also investigating community composition, as the effects of stressors on food selection may explain observed shifts in bacterial communities. Exposure to increased salinity significantly altered the feeding behavior of the flagellates by increasing the selection against *Microbacterium lacusdiani* (Fig. 2). This was unexpected, as previous studies suggest that protistan predators become less selective when facing environmental changes [41]. Interestingly, pre-stress feeding behavior was fully restored within 48 h after stressor removal (Fig. 2). The fast recovery of the feeding behavior suggests that the observed changes may not result from the shifts in the predator population but rather from physiological acclimatization and/or changes in the characteristics of the food source in response to increased salinity. High salt concentrations can alter the surface charge of both gram-negative and gram-positive bacteria; however, this effect may vary between and within bacterial groups [85, 86]. As previously mentioned, the food selection of HNFs can be influenced by the physicochemical surface properties of their prey, i.e. particles with lower surface charges tend to be ingested more frequently than those with higher charges [30, 87].

The salt treatment also affected the microbial community composition, causing community composition in the salt and combination treatments to significantly differ from the control and temperature treatments (Fig. 3C and D). Our results indicate that both prokaryotic and the microeukaryotic community were affected by the salt treatment. However, the effects were more pronounced in the microeukaryotic community than in the bacterial communities. While previous studies indicated that bacteria are more sensitive than microeukaryotes when exposed to heat stress [88, 89], our results align with previous research in suggesting that microeukaryotes may be more sensitive than bacteria to salinization [90]. This highlights that the sensitivity of prokaryotic and microeukaryotic communities may vary depending on the specific stressor.

The rapid increase of Chrysophyceae and Choanoflagellida in the salt and combination treatments, consistent with previous research suggesting their tolerance of elevated salinity [91–93], indicates a selective advantage of these groups under salinization. Since bacterial communities are shaped by protist predation, significant changes in the relative abundance and food selection of bacterivorous protists can alter the composition of the prokaryotic community [23, 24, 26]. In our experiments, Actinomycetota was the third most abundant phylum across all three replicates prior to stressor addition and maintained this position in both the control and temperature treatments. In contrast, under salinization, the relative abundance of Actinomycetota decreased, being replaced by Cyanobacteria as the third most abundant phylum in the salt and combination treatments. This aligns with previous studies demonstrating that Actinomycetota in freshwater habitats are negatively affected by increasing salt concentration [90, 94]. On the other hand, it also indicates that this negative effect on growth counterbalanced the positive effect of reduced grazing pressure by HNFs. This suggests that the effect of stressors on selective feeding may strongly affect community composition, possibly also altering ecosystem functions, when bacterial taxa are concerned which are less sensitive to the stressor and thus may overgrow their competitors due to such relaxed grazing.

Interestingly, genera within the same phylum responded differently to stress (Fig. 5). The decline of OTUs related to *Microbacterium lausdiani* was more pronounced in control and temperature treatments than in salt and combination treatments, likely due to reduced grazing pressure by HNFs under salinization. Stress responses also varied within the Pseudomonadota. Despite the overall increase of Pseudomonadota across all treatments, the relative abundance of *Limnohabitans* decreased in the salt and combination treatments, probably due to increased grazing pressure when exposed to increased salinity (Fig. 5). In contrast, other Pseudomonadota, such as *Hydrogenophaga* and *Pseudomonas,* showed higher relative abundance when exposed to increased salt concentration compared to the mesocosms where salinity remained unchanged (Fig. 5), aligning with previous studies that demonstrated that Pseudomonadota are positively affected by increases in salinity [90, 95]. Our results highlight that stress responses can vary strongly among different groups and can be masked by turnovers of closely related taxa.

The different sampling times for the three replicates resulted in significant differences in the starting community. However, we observed similar stressor-induced effects despite clearly distinct microbial communities at the beginning of each replicate (Fig. 4). Additionally, temporal effects typical of such mesocosm experiments [43, 96, 97] shaped the microbial communities in our systems. Over time, prokaryotic communities became more similar across the three replicates, with the same pattern observed for microeukaryotes (Fig. 3), which was likely due to the experimental setup favoring a specific community. In many experiments in microbial ecology, a high variation between replicates is diminished by using subsamples of one bulk sample for replication or by taking replicate samples at the same time. While this approach provides replication for that particular sample or time point, it limits generalization beyond the specific instance. Ideally, generalizations about the broader habitat (e.g. a lake or stream) would be based on samples collected at independent time points [98], which is often challenging due to factors such as the high costs and resources needed for repeated large-scale experiments or when studying rare natural events [99, 100]. By conducting three mesocosm experiments using water and sediment from the same stream but sampled at different time points, we accounted for the natural variation in freshwater habitats. This approach enabled better validation of effect size and allowed for broader generalization of the results, irrespective of differences in the starting community [98].

Overall, this study demonstrates that environmental changes can significantly impact the food selection of HNFs. Our findings provide valuable insights into the feeding behavior of HNFs in response to short-term salinization, a crucial first step in understanding the overall impact of such disturbances on HNF grazing and potential cascading effects on their prey populations. Further studies are required to assess whether the observed community response is primarily driven by dominant taxa or a uniform reaction from most HNFs and to explore the influence of larger protists on shaping HNF communities under changing environmental conditions. Finally, investigating long-term effects of such abiotic disturbances in different freshwater bodies is essential for determining the broader impacts of environmental change on microbial communities in freshwater ecosystems.

## Supplementary Material

Supplements_revised_ycaf049

16S_OTU_full_ycaf049

18S_OTU_full_ycaf049

## Data Availability

Amplicon sequence data generated and analysed during the current study have been submitted to the NCBI SRA repository, accession number PRJNA1178677. The microscopy images generated and analysed during the current study are available from the corresponding author on reasonable request.

## References

[1] Trzcinski MK, Srivastava DS, Corbara B et al. The effects of food web structure on ecosystem function exceeds those of precipitat*ion*. *J Anim Ecol* 2016;85:1147–60. 10.1111/1365-2656.12538 27120013

[2] Domingues CD, da Silva LHS, Rangel LM et al. Microbial food-web drivers in tropical reservoirs. *Microb Ecol* 2016;73:505–20. 10.1007/s00248-016-0899-1 27900461

[3] Guo P, Li C, Liu J et al. Predation has a significant impact on the complexity and stability of microbial food webs in subalpine lakes. *Microbiol Spectr* 2023;11:e02411–23. 10.1128/spectrum.02411-23 37787559 PMC10714739

[4] Jane S, Hansen G, Kraemer B et al. Widespread deoxygenation of temperate lakes. *Nature* 2021;594:66–70. 10.1038/s41586-021-03550-y 34079137

[5] Woolway R, Sharma S, Smol J. Lakes in hot water: the impacts of a changing climate on aquatic ecosystems. *Bioscience* 2022;72:1050–61. 10.1093/biosci/biac052 36325103 PMC9618276

[6] Woolway R, Jennings E, Shatwell T et al. Lake heatwaves under climate change. *Nature* 2021;589:402–7. 10.1038/s41586-020-03119-1 33473224

[7] Cañedo-Argüelles M, Kefford B, Schäfer R. Salt in freshwaters: causes, effects and prospects—introduction to the theme issue. *Philos Trans R Soc B* 2019;374:20180002. 10.1098/rstb.2018.0002 PMC6283966 30509904

[8] Hintz WD, Relyea RA. A review of the species, community, and ecosystem impacts of road salt salinisation in fresh waters. *Freshw Biol* 2019;64:1081–97. 10.1111/fwb.13286

[9] Gombert P, Sracek O, Koukouzas N et al. An overview of priority pollutants in selected coal mine discharges in Europe. *Mine Water Environ* 2019;38:16–23. 10.1007/s10230-018-0547-8

[10] Sun X, Arnott SE. Interactive effects of increased salinity and heatwaves on freshwater zooplankton communities in simultaneous and sequential treatments. *Freshw Biol* 2022;67:1604–17. 10.1111/fwb.13964

[11] Hao B, Roejkjaer AF, Wu H et al. Responses of primary producers in shallow lakes to elevated temperature: a mesocosm experiment during the growing season of Potamogeton crispus. *Aquat Sci* 2018;80:34. 10.1007/s00027-018-0585-0

[12] Thomson AH, Manoylov KM. Algal community dynamics within the Savannah River estuary, Georgia under anthropogenic stress. *Estuar Coasts* 2019;42:1459–74. 10.1007/s12237-019-00579-1

[13] Engloner AI, Vargha M, Kós P et al. Planktonic and epilithic prokaryota community compositions in a large temperate river reflect climate change related seasonal shifts. *PLoS One* 2023;18:e0292057. 10.1371/journal.pone.0292057 37733803 PMC10513243

[14] Machado K, Antunes A, Targueta C et al. DNA metabarcoding reveals the responses of prokaryotes and eukaryotes microbiota to warming: are the patterns similar between taxonomic and trophic groups? *Ecol Indic* 2020;115:106452. 10.1016/j.ecolind.2020.106452

[15] von Alvensleben N, Magnusson M, Heimann K. Salinity tolerance of four freshwater microalgal species and the effects of salinity and nutrient limitation on biochemical profiles. *J Appl Phycol* 2016;28:861–76. 10.1007/s10811-015-0666-6

[16] Fournier IB, Lovejoy C, Vincent WF. Changes in the community structure of under-ice and open-water microbiomes in urban lakes exposed to road salts. *Front Microbiol* 2021;12:660719. 10.3389/fmicb.2021.660719 33868217 PMC8044900

[17] Li J, Ye W, Wei D et al. System performance and microbial community succession in a partial nitrification biofilm reactor in response to salinity stress. *Bioresource Tech* 2018;270:512–8. 10.1016/j.biortech.2018.09.068 30248650

[18] Nuy JK, Lange A, Beermann AJ et al. Responses of stream microbes to multiple anthropogenic stressors in a mesocosm study. *Sci Tot Environ* 2018;633:1287–301. 10.1016/j.scitotenv.2018.03.077 29758882

[19] Phillips K, Godwin C, Cotner J. The effects of nutrient imbalances and temperature on the biomass stoichiometry of freshwater bacteria. *Front Microbiol* 2017;8:1692. 10.3389/fmicb.2017.01692 28943865 PMC5596061

[20] Zohary T, Flaim G, Sommer U. Temperature and the size of freshwater phytoplankton. *Hydrobiologia* 2020;848:143–55. 10.1007/s10750-020-04246-6

[21] Franzè G, Menden-Deuer S. Common temperature-growth dependency and acclimation response in three herbivorous protists. *Mar Ecol Prog Ser* 2020;634:1–13. 10.3354/meps13200

[22] Atkinson D, Ciotti B, Montagnes D. Protists decrease in size linearly with temperature: ca. 2.5% °C−1. *Proc R Soc Lond B Biol Sci* 2003;270:2605–11. 10.1098/rspb.2003.2538 PMC1691543 14728784

[23] Sherr EB, Sherr BF. Significant of predation by protists in aquatic microbial food webs. *Antonie Van Leewenhoek* 2002;81:293–308. 10.1023/A:1020591307260 12448728

[24] Bochdansky AB, Clouse MA. New tracer to estimate community predation rates of phagotrophic protists. *Mar Ecol Prog Ser* 2015;524:55–69. 10.3354/meps11209

[25] Šimek K, Chrzanowski TH. Direct and indirect evidence of size-selective grazing on pelagic bacteria by freshwater nanoflagellates. *Appl Environ Microbiol* 1992;58:3715–20. 10.1128/aem.58.11.3715-3720.1992 16348811 PMC183165

[26] Batani G, Pérez G, Martínez de la Escalera G et al. Competition and protist predation are important regulators of riverine bacterial community composition and size distribution. *J Freshw Ecol* 2016;31:609–23. 10.1080/02705060.2016.1209443

[27] Selph KE, Landry MR, Laws EA. Heterotrophic nanoflagellate enhancement of bacterial growth through nutrient remineralization in chemostat culture. *Aquat Microb Ecol* 2003;32:23–37. 10.3354/ame032023

[28] Weinbauer M, Horňák K, Jezbera J et al. Synergistic and antagonistic effects of viral lysis and protistan grazing on bacterial biomass, production and diversity. *Environ Microbiol* 2007;9:777–88. 10.1111/j.1462-2920.2006.01200.x 17298376

[29] Das N, Pandey A. Role of nanoplanktons in marine food-webs. *Int Lett Nat Sci* 2015;43:38–47.

[30] Matz C, Boenigk J, Arndt H et al. Role of bacterial phenotypic traits in selective feeding of the heterotrophic nanoflagellate *Spumella sp*. *Aquat Microb Ecol* 2002;27:137–48. 10.3354/ame027137

[31] Shannon SP, Chrzanowski TH, Grover JP. Prey food quality affects flagellate ingestion rates. *Microb Ecol* 2006;53:66–73. 17186152 10.1007/s00248-006-9140-y

[32] Boenigk J, Matz C, Jürgens K et al. The influence of preculture conditions and food quality on the ingestion and digestion process of three species of heterotrophic nanoflagellates. *Microb Ecol* 2001;42:168–76. 12024279 10.1007/s002480000116

[33] Boenigk J, Matz C, Jürgens K et al. Food concentration-dependent regulation of food selectivity of interception-feeding bacterivorous nanoflagellates. *Aquat Microb Ecol* 2002;27:195–202. 10.3354/ame027195

[34] Tophøj J, Wollenberg R, Sondergaard T et al. Feeding and growth of the marine heterotrophic nanoflagellates, *Procryptobia sorokini* and *Paraphysomonas imperforata* on a bacterium, *Pseudoalteromonas sp*. with an inducible defence against grazing. *PLoS One* 2018;13:e0195935. 10.1371/journal.pone.0195935 29652905 PMC5898755

[35] Sintes E, Giorgio P. Feedbacks between protistan single-cell activity and bacterial physiological structure reinforce the predator/prey link in microbial food webs. *Front Microbiol* 2014;5:453. 25250018 10.3389/fmicb.2014.00453 PMC4155813

[36] Vázquez-Domínguez E, Vaqué D, Gasol JM. Temperature effects on the heterotrophic bacteria, heterotrophic nanoflagellates, and microbial top predators of the NW Mediterranean. *Aquat Microb Ecol* 2012;67:107–21. 10.3354/ame01583

[37] Zingel P, Cremona F, Nõges T et al. Effects of warming and nutrients on the microbial food web in shallow lake mesocosms. *Eur J Protistol* 2018;64:1–12. 10.1016/j.ejop.2018.03.001 29621651

[38] DeVilbiss S, Steele M, Brown B et al. Stream bacterial diversity peaks at intermediate freshwater salinity and varies by salt type. *Sci Total Environ* 2022;840:156690. 10.1016/j.scitotenv.2022.156690 35714745

[39] Tammert H, Kivistik C, Kisand V et al. Resistance of freshwater sediment bacterial communities to salinity disturbance and the implication for industrial salt discharge and climate change-based salinization. *Front Microbiomes* 2023;2:1232571. 10.3389/frmbi.2023.1232571 PMC12993645 41853338

[40] Bock C, Jensen M, Forster D et al. Factors shaping community patterns of protists and bacteria on a European scale. *Environ Microbiol* 2020;22:2243–60. 10.1111/1462-2920.14992 32202362

[41] Staniczenko PPA, Lewis OT, Jones NS et al. Structural dynamics and robustness of food webs. *Ecol Lett* 2010;13:891–9. 10.1111/j.1461-0248.2010.01485.x 20482578

[42] Velasco J, Gutiérrez-Cánovas C, Botella-Cruz M et al. Effects of salinity changes on aquatic organisms in a multiple stressor context. *Philos Trans R Soc B* 2018;374:20180011. 10.1098/rstb.2018.0011 PMC6283958 30509913

[43] Graupner N, Röhl O, Jensen M et al. Effects of short-term flooding on aquatic and terrestrial microeukaryotic communities: a mesocosm approach. *Aquat Microb Ecol* 2017;80:257–72. 10.3354/ame01853

[44] Suzuki KI, Hamada M, Genus I. Microbacterium. In: Goodfellow M., Kämpfer P., Busse H.-J. et al. (eds.), Bergey’s Manual of Systematic Bacteriology, Vol. 5, 2nd edn. New York, NY: Springer, 2012, 814–52.

[45] Kasalický V, Jezbera J, Hahn M et al. The diversity of the *Limnohabitan*s Genus, an important group of freshwater bacterioplankton, by characterization of 35 isolated strains. *PLoS One* 2013;8:e58209. 10.1371/journal.pone.0058209 23505469 PMC3591437

[46] Ouyang L, Chen H, Liu X et al. Characteristics of spatial and seasonal bacterial community structures in a river under anthropogenic disturbances. *Environ Pollut* 2020;264:114818. 10.1016/j.envpol.2020.114818 32559870

[47] Zhurlov O, Nemtseva N, Grudinin D et al. Bacterial community composition in the rivers of the Novaya Sibir Island. *Microbiol* 2019;2019:499–504.

[48] Najjar P, Pfaffl M, Ouaini N et al. Water and sediment microbiota diversity in response to temporal variation at the outlet of the Ibrahim River (Lebanon). *Environ Monit Assess* 2020;192:1–11. 10.1007/s10661-020-8139-z 32107647

[49] Hahn MW, Lundsdorf H, Wu QL et al. Isolation of novel ultramicrobacteria classified as actinobacteria from five freshwater habitats in Europe and Asia. *Appl Environ Microb* 2003;69:1442–51. 10.1128/AEM.69.3.1442-1451.2003 PMC150105 12620827

[50] Piwosz K, Mukherjee I, Salcher MM et al. CARD-FISH in the sequencing era: opening a new universe of protistan ecology. *Front Microbiol* 2021;12:640066. 10.3389/fmicb.2021.640066 33746931 PMC7970053

[51] Buchner D . Sample Preparation and Lysis of Homogenized Malaise Trap Samples v1. London, UK: Springer Nature, 2022. 10.17504/protocols.io.dm6gpjrmjgzp/v1

[52] Buchner D . Guanidine-Based DNA Extraction with Silica-Coated Beads or Silica Spin Columns v1. London, UK: Springer Nature, 2022. 10.17504/protocols.io.eq2ly73mmlx9/v1

[53] Buchner D . PCR Cleanup and Size Selection with Magnetic Beads v2. London, UK: Springer Nature, 2022. 10.17504/protocols.io.36wgqj45xvk5/v2

[54] Caporaso JG, Lauber CL, Walters WA et al. Global patterns of 16S rRNA diversity at a depth of millions of sequences per sample. *Proc Natl Acad Sci USA* 2011;108:4516–22. 10.1073/pnas.1000080107 20534432 PMC3063599

[55] Amaral-Zettler LA, McCliment EA, Ducklow HW et al. A method for studying protistan diversity using massively parallel sequencing of V9 hypervariable regions of small-subunit ribosomal RNA genes. *PLoS One* 2009;4:e6372. 10.1371/journal.pone.0006372 19633714 PMC2711349

[56] Deep A, Bludau D, Welzel M et al. Natrix2—improved amplicon workflow with novel Oxford Nanopore Technologies support and enhancements in clustering, classification and taxonomic databases. *Metabarcoding Metagenomics* 2023;7:e109389. 10.3897/mbmg.7.109389

[57] Masella AP, Bartram AK, Truszkowski JM et al. PANDAseq: paired-end assembler for illumina sequences. *BMC Bioinformatics* 2012;13:31. 10.1186/1471-2105-13-31 22333067 PMC3471323

[58] Fu L, Niu B, Zhu Z et al. CD-HIT: accelerated for clustering the next-generation sequencing data. *Bioinformatics* 2012;28:3150–2. 10.1093/bioinformatics/bts565 23060610 PMC3516142

[59] Rognes T, Flouri T, Nichols B et al. VSEARCH: a versatile open source tool for metagenomics. *PeerJ* 2016;4:e2584. 10.7717/peerj.2584 27781170 PMC5075697

[60] Lange A, Jost S, Heider D et al. AmpliconDuo: a split-sample filtering protocol for high-throughput amplicon sequencing of microbial communities. *PLoS One* 2015;10:e0141590. 10.1371/journal.pone.0141590 26523925 PMC4629888

[61] Mahé F, Czech L, Stamatakis A et al. Swarm v3: towards tera-scale amplicon clustering. *Bioinformatics* 2022;38:267–9. 10.1093/bioinformatics/btab493 PMC8696092 34244702

[62] Schloss PD, Westcott SL, Ryabin T et al. Introducing mothur: open-source, platform-independent, community-supported software for describing and comparing microbial communities. *Appl Environ Microbiol* 2009;75:7537–41. 10.1128/AEM.01541-09 19801464 PMC2786419

[63] Quast C, Pruesse E, Yilmaz P et al. The SILVA ribosomal RNA gene database project: improved data processing and web-based tools. *Nucleic Acids Res* 2013;41:D590–6. 10.1093/nar/gks1219 23193283 PMC3531112

[64] Guillou L, Bachar D, Audic S et al. The protist ribosomal reference database (PR2): a catalog of unicellular eukaryote small sub-unit rRNA sequences with curated taxonomy. *Nucleic Acids Res* 2013;41:D597–604. 10.1093/nar/gks1160 23193267 PMC3531120

[65] Mahé F . Mumu: Post-Clustering Curation Tool for Metabarcoding Data. San Francisco, USA: GitHub, 2022. (v.0.01). https://github.com/frederic-mahe/mumu

[66] PositTeam. RStudio: Integrated Development Environment for R. Boston, USA: Posit Software, 2024. http://www.posit.co/.

[67] McMurdie PJ, Holmes S. Phyloseq: an R package for reproducible interactive analysis and graphics of microbiome census data. *PLoS One* 2013;8:e61217. 10.1371/journal.pone.0061217 23630581 PMC3632530

[68] Love MI, Huber W, Anders S. Moderated estimation of fold change and dispersion for RNA-seq data with DESeq2. *Genome Biol* 2014;15:1–21. 10.1186/s13059-014-0550-8 PMC4302049 25516281

[69] Wickham H . ggplot2: Elegant Graphics for Data Analysis. NewYork: Springer Publishing, 2016. 10.1007/978-3-319-24277-4

[70] Teunisse GM . Fantaxtic-Nested Bar Plots for Phyloseq Data. San Francisco, USA: GitHub, 2022. (R package version 0.2.1). https://github.com/gmteunisse/Fantaxtic

[71] Barnett DJ, Arts IC, Penders J. microViz: an R package for microbiome data visualization and statistics. *J Open Source Softw* 2021;6:3201. 10.21105/joss.03201

[72] Oksanen J, Simpson G, Blanchet F et al. Vegan: Community Ecology Package. Vienna, Austria: R Foundation for Statistical Computing, 2024. R package version 2.6-8. https://CRAN.R-project.org/package=vegan

[73] Wickham H, Francois R, Henry L et al. Dplyr: A Grammar of Data Manipulation. Vienna, Austria: R Foundation for Statistical Computing, 2024. R package version 1.1.4. https://CRAN.R-project.org/package=dplyr

[74] Dinno A . Dunn.Test: Dunn’s Test of Multiple Comparisons Using Rank Sums. Vienna, Austria: R Foundation for Statistical Computing, 2024. R package version 1.3.6. https://CRAN.R-project.org/package=dunn.test

[75] Pernthaler A, Pernthaler J, Amann R. Sensitive multicolor fluorescence *in situ* hybridization for the identification of environmental microorganisms. In: Kowalchuk G., de Bruijn F.J., Head I.M. et al. (eds.), Molecular Microbial Ecology Manual, 2nd 3.11 Ed. Dordrecht, Boston, London: Kluwer Academic Publishers, 2004, 711–26.

[76] Šimek K, Pernthaler J, Weinbauer MG et al. Changes in bacterial community composition and dynamics and viral mortality rates associated with enhanced flagellate grazing in a mesoeutrophic reservoir. *Appl Environ Microbiol* 2001;67:2723–33. 10.1128/AEM.67.6.2723-2733.2001 11375187 PMC92931

[77] Roller C, Wagner M, Amann R et al. *In situ* probing of gram-positive bacteria with high DNA G+C content with 23S rRNA-targeted oligonucleotides. *Microbiol* 1994;140:2849–58. 10.1099/00221287-140-10-2849 8000548

[78] Schindelin J, Arganda-Carreras I, Frise E et al. Fiji: an open-source platform for biological-image analysis. *Nat Methods* 2012;9:676–82. 10.1038/nmeth.2019 22743772 PMC3855844

[79] Katrukha E . ComDet Plugin for ImageJ. San Francisco, USA: GitHub, 2021. (v0.5.5). https://github.com/UU-cellbiology/ComDet

[80] Takahashi T . Applicability of automated cell counter with a chlorophyll detector in routine management of microalgae. *Sci Rep* 2018;8:4967. 10.1038/s41598-018-23311-8 29563559 PMC5862891

[81] Chesson J . The estimation and analysis of preference and its relationship to foraging models. *Ecol* 1983;64:1297–304. 10.2307/1937838

[82] Wickham H, Bryan J. Readxl: Read Excel Files. Vienna, Austria: R Foundation for Statistical Computing, 2023. (R package version 1.4.3). https://CRAN.R-project.org/package=readxl

[83] Wickham H, Henry L. Purrr: Functional Programming Tools. Vienna, Austria: R Foundation for Statistical Computing, 2023. (R package version 1.0.2). https://CRAN.R-project.org/package=purrr

[84] Rocca JD, Yammine A, Simonin M et al. Protist predation influences the temperature response of bacterial communities. *Front Microbiol* 2022;13:847964. 10.3389/fmicb.2022.847964 35464948 PMC9022080

[85] Hong Y, Brown DG. Electrostatic behavior of the charge-regulated bacterial cell surface. *Langmuir* 2008;24:5003–9. 10.1021/la703564q 18363414

[86] Wyness A, Paterson D, Defew E et al. The role of zeta potential in the adhesion of E. coli to suspended intertidal sediments. *Water Res* 2018;142:159–66. 10.1016/j.watres.2018.05.054 29870949

[87] Hammer A, Grüttner C, Schumann R. The effect of electrostatic charge of food particles on capture efficiency by Oxyrrhis marina Dujardin (dinoflagellate). *Protist* 1999;150:375–82. 10.1016/S1434-4610(99)70039-8 10714772

[88] Thakur MP, van der Putten WH, Apon F et al. Resilience of rhizosphere microbial predators and their prey communities after an extreme heat event. *Funct Ecol* 2021;35:216–25. 10.1111/1365-2435.13696

[89] Fang G, Yu H, Sheng H et al. Comparative analysis of microbial communities between water and sediment in Laoshan Bay marine ranching with varied aquaculture activities. *Mar Pollut Bull* 2021;173:112990. 10.1016/j.marpolbul.2021.112990 34634629

[90] Gagnon J-C, Astorg L, Derry A et al. Response of prokaryotic communities to freshwater salinization. *Appl Microbiol* 2022;2:330–46.

[91] Astorg L, Gagnon J-C, Lazar CS et al. Effects of freshwater salinization on a salt-naïve planktonic eukaryote community. *Limnol Oceanogr Lett* 2023;8:38–47. 10.1002/lol2.10229

[92] Misiak K, Dunlap KJ, Rodriguez SS et al. Tolerance of a choanoflagellate to environmental stressors. *FASEB J* 2008;22:648.21-648.21. 10.1096/fasebj.22.1_supplement.648.21

[93] Schiwitza S, Arndt H, Nitsche F. First description of an euryoecious acanthoecid choanoflagellate species, *Enibas tolerabilis gen. et sp. nov*. from a salar in the Chilean Andes based on morphological and transcriptomic data. *Eur J Protistol* 2019;67:106–13. 10.1016/j.ejop.2018.11.004 30572146

[94] Lew S, Glińska-Lewczuk K, Burandt P et al. Salinity as a determinant structuring microbial communities in Coastal Lakes. *Int J Environ Res Public Health* 2022;19:4592. 10.3390/ijerph19084592 35457457 PMC9028135

[95] Newton RJ, McLellan SL. A unique assemblage of cosmopolitan freshwater bacteria and higher community diversity differentiate an urbanized estuary from oligotrophic. Lake Michigan. *Front Microbiol* 2015;6:1028. 26483766 10.3389/fmicb.2015.01028 PMC4586452

[96] Sieber G, Drees F, Shah M et al. Exploring the efficacy of metabarcoding and non-target screening for detecting treated wastewater. *Sci Total Environ* 2023;903:167457. 10.1016/j.scitotenv.2023.167457 37777125

[97] Stach TL, Sieber G, Shah M et al. Temporal disturbance of a model stream ecosystem by high microbial diversity from treated wastewater. *Microbiology* 2023;12:e1347. 10.1002/mbo3.1347 PMC10012233 37186231

[98] Beentjes KK, Speksnijder AGCL, Schilthuizen M et al. The effects of spatial and temporal replicate sampling on eDNA metabarcoding. *PeerJ* 2019;7:e7335. 10.7717/peerj.7335 31388472 PMC6662575

[99] Nikinmaa M, Celander M, Tjeerdema R. Replication in aquatic biology: the result is often pseudoreplication. *Aquat Toxicol* 2012;15:116–7. 10.1016/S0166-445X(12)00141-5 22520022

[100] Davies G, Gray A. Don’t let spurious accusations of pseudoreplication limit our ability to learn from natural experiments (and other messy kinds of ecological monitoring). *Ecol Evol* 2015;5:5295–304. 10.1002/ece3.1782 30151132 PMC6102510

