# Supplementary material for: The impact of elevated temperature and salinity on microbial communities and food selectivity in heterotrophic nanoflagellates in the Boye River
Source: ISME Commun. 2025 Mar 21;5(1):ycaf049. doi: 10.1093/ismeco/ycaf049 (PMC11976726; doi:10.1093/ismeco/ycaf049)
Supplement: Supplements_revised_ycaf049 [file supplements_revised_ycaf049.docx]

**Supplementary information for**

**The impact of elevated temperature and salinity on microbial communities and food selectivity in heterotrophic nanoflagellates in the Boye River**

Lisa Boden^1^, Dana Bludau^1,2,3^, Guido Sieber^1,2^, Aman Deep^1,3^, Daria Baikova^4^, Gwendoline M. David^5^, Una Hadžiomerović^2,4^, Tom L. Stach^2,6^, & Jens Boenigk^1,2^

*^1^Department Biodiversity, University of Duisburg–Essen, Essen, Germany*

*^2^Center for Water and Environmental Research, University of Duisburg–Essen, Essen, Germany*

*^3^Department of Engineering and Natural Sciences, Westphalian University of Applied Sciences,*

*Recklinghausen, Germany*

*^4^Department* *Environmental Microbiology and Biotechnology, University of Duisburg–Essen, Essen, Germany*

*^5^Department of Plankton and Microbial Ecology, Leibniz Institute of Freshwater Ecology and Inland Fisheries (IGB), Stechlin, Germany*

*^6^Environmental Metagenomics, Research Center One Health Ruhr, University Alliance Ruhr, University of Duisburg–Essen, Essen, Germany*

**Corresponding authors**

Lisa Boden^1^

lisa.boden@uni-due.de


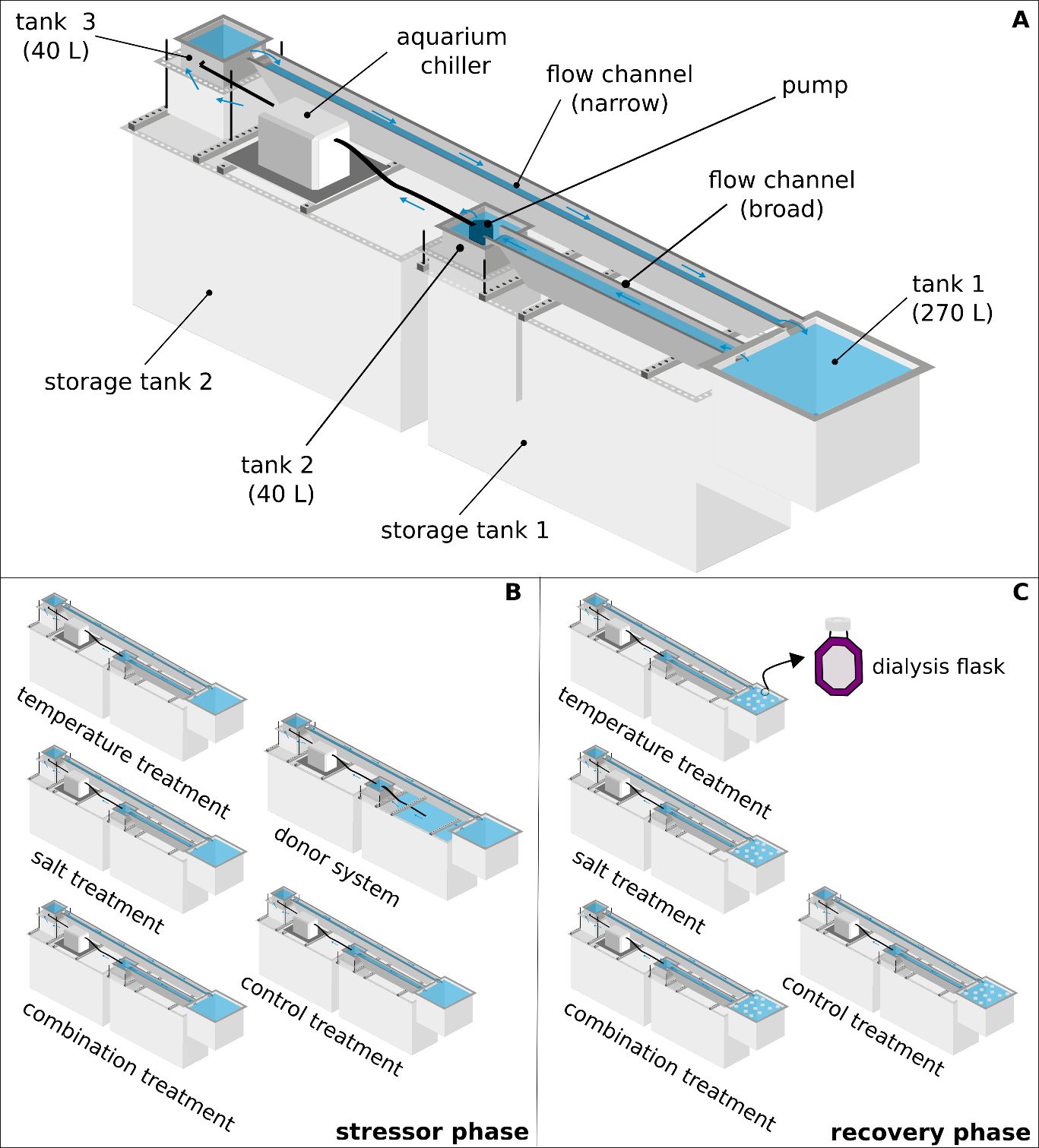


**Figure S1**. Schematic of an AquaFlow mesocosm and full experimental setup. (A) Each mesocosm consists of three steel tanks, a narrow flow channel (5 cm width, 4 m length) connecting tanks 1 and 2 and a broader flow channel (10 cm width, 2 m length) connecting tanks 2 and 3. A pump and an aquarium chiller-controlled flow velocity and water temperature. (B) During the stressor phase, 4 identical mesocosms each exposed to a different stressor treatment (control, increased temperature, increased salinity, combination of both stressors) were used. The donor system was altered to circulate more water than the other 4 and was run under control conditions. (C) After stressor removal, dialysis flasks containing previously stressed water from each mesocosm were added to tank 3 of each corresponding mesocosm. The donor system was removed after stressor release.

**Table S1**. Reagents and cycling conditions of the first and the second PCR for 16S and 18S rRNA gene amplification. 20 cycles were used for the first PCR and 25 cycles were used for the second PCR.

| **PCR reagent** | **1^st^ PCR** | **2^nd^ PCR** |
| --- | --- | --- |
| Multiplex Master Mix | 5 µl | 5 µl |
| Forward Primer | 200 nM | 100 nM |
| Reverse Primer | 200 nM | 100 nM |
| DNA | 1 µl | 2 µl |
| Nuclease-free H_2_O | 3.6 µl | / |
| Coral Load | / | 1 µl |
| **Cycle conditions** |  |  |
| Initial denaturation | 5 min – 95 °C | 5 min – 95 °C |
| Denaturation | 30 s – 95 °C | 30 s – 95 °C |
| Annealing | 90 s - primer specific | 90 s – 61 °C |
| Elongation | 30s – 72 °C | 30s – 72 °C |
| Final Elongation | 10 min – 68 °C | 10 min – 68 °C |

**Table S2. Python script used to subtract contamination detected in negative controls from sample read counts.**

| **##This script is to subtract the maximum read counts of OTUs found in the negative controls from the corresponding read counts in each sample.**  **## usage: python script.py full_table out_table**  import pandas as pd  import sys  file_path = sys.argv[1]  df = pd.read_csv(file_path)  column_names = df.columns.tolist()  column_names = column_names[2:-1]  cleaned_column_names = []  taxonomy_df=df["taxonomy"]  cleaned_column_names = [column_name[:-2] if column_name.endswith("_A") or column_name.endswith("_B") else column_name for column_name in column_names]  uniq_col_names=list(set(cleaned_column_names))  **# Iterate through each sample, calculate sum, and create a new column**  for sample in uniq_col_names:  a_col = f'{sample}_A'  b_col = f'{sample}_B'  df[sample] = df.apply(lambda row: row[a_col] + row[b_col] if row[a_col] * row[b_col] != 0 else 0, axis=1)  # Create a new DataFrame with only 'seqid' and the summed columns  output_columns = ['seqid'] + cleaned_column_names  output_df = df[output_columns]  output_df = output_df.loc[:, ~output_df.columns.duplicated()]  # **separate each experiment**  pattern = r'AF1'  af1 = output_df.filter(regex=pattern)  pat= r'NC'  nc_af1 = af1.filter(regex=pat)  af1_row=nc_af1.max(axis=1)  af1_max = pd.DataFrame({'max': af1_row})  af1_df3=pd.concat([af1,af1_max],axis=1)  # **Convert numeric columns in the original DataFrame to numeric type**  af1_df_numeric = af1.select_dtypes(include=[int, float])  # Subtract the maximum values from numeric columns (excluding the first column) # put 0 if negative  af1_subtracted_df = af1_df_numeric.sub(af1_max['max'], axis=0).applymap(lambda x: 0 if x < 0 else x)  pattern = r'AF2'  af2 = output_df.filter(regex=pattern)  pat= r'NC'  nc_af2 = af2.filter(regex=pat)  af2_row=nc_af2.max(axis=1)  af2_max = pd.DataFrame({'max': af2_row})  af2_df3=pd.concat([af2,af2_max],axis=1)  af2_df_numeric = af2.select_dtypes(include=[int, float])  **# Subtract the maximum values from numeric columns (excluding the first column)**  **# put 0 if negative**  af2_subtracted_df = af2_df_numeric.sub(af2_max['max'], axis=0).applymap(lambda x: 0 if x < 0 else x)  pattern = r'AF3'  af3 = output_df.filter(regex=pattern)  pat= r'NC_'  nc_af3 = af3.filter(regex=pat)  af3_row=nc_af3.max(axis=1)  af3_max = pd.DataFrame({'max': af3_row})  af3_df3=pd.concat([af3,af3_max],axis=1)  af3_df_numeric = af3.select_dtypes(include=[int, float])  # Subtract the maximum values from numeric columns (excluding the first column) # put 0 if negative  af3_subtracted_df = af3_df_numeric.sub(af3_max['max'], axis=0).applymap(lambda x: 0 if x < 0 else x)  **# Concatenate the first column and non-numeric columns with the subtracted DataFrame**  final_result = pd.concat([output_df['seqid'], af1_subtracted_df, af2_subtracted_df, af3_subtracted_df, taxonomy_df], axis=1)  final_result.T.drop_duplicates().T  **#remove all rows with no values**  non_null_counts = final_result.count(axis=1)  filtered_df = final_result[non_null_counts > 2]  **## remove NC columns**  pattern=r'D\|tax\|seq'  samples_df = filtered_df.filter(regex=pattern)  #filtered_df.to_csv(sys.argv[2], index=False)  samples_df.to_csv(sys.argv[2], index=False) |
| --- |

**Table S3**. Oligonucleotide probes used for CARD-FISH

| **Probe** | **Sequence (5' to 3')** | **Specificity** | **Formamide** | **rRNA binding site** |
| --- | --- | --- | --- | --- |
| *HCG69a* | TATAGTTACCACCGCCGT | *Actinobacteria* | 25 % | 23S, 1901-1918 |
| *R-BT065* | GTTGCCCCCTCTACCGTT | R-BT lineage | 35 % | 16S, 65-82 |

**Table S4.** Total counts of flagellates, ingested bacteria, and ingested beads, along with the average counts of bacteria and beads per sample. T0 represents samples fixed immediately after food source addition, while T12 represents samples fixed 12 minutes after addition.

| **Sample** | **Flagellates** | ***Limnohabitans*** | ***Limn._ingested*** | **Beads** | **Beads_ingested** | **Flagellates** | ***Microbacterium*** | ***Microbac._ingested*** | **Beads** | **Beads_ingested** |
| --- | --- | --- | --- | --- | --- | --- | --- | --- | --- | --- |
| **Experiment 1_ Control_Day 1 (pre-stress)** | | | | | | | | | | |
| replicate 1_T0 | 24 | 404 | 8 | 394 | 0 | 36 | 472 | 16 | 379 | 0 |
| replicate_ 1_T12 | 39 |  | 33 |  | 7 | 26 |  | 22 |  | 5 |
| replicate_ 2_T0 | 39 | 237 | 18 | 442 | 0 | 70 | 266 | 19 | 390 | 0 |
| replicate_ 2_T12 | 37 |  | 28 |  | 4 | 65 |  | 45 |  | 3 |
| replicate_ 3_T0 | 20 | 166 | 6 | 320 | 0 | 37 | 99 | 15 | 268 | 0 |
| replicate_ 3_T12 | 37 |  | 29 |  | 11 | 30 |  | 18 |  | 8 |
| **Experiment 2_ Control_Day 1 (pre-stress)** | | | | | | | | | | |
| replicate_ 1_T0 | 42 | 264 | 15 | 271 | 0 | 47 | 283 | 17 | 351 | 0 |
| replicate_ 1_T12 | 55 |  | 37 |  | 4 | 35 |  | 20 |  | 3 |
| replicate_ 2_T0 | 55 | 242 | 25 | 334 | 0 | 53 | 274 | 22 | 350 | 0 |
| replicate_ 2_T12 | 50 |  | 34 |  | 5 | 50 |  | 34 |  | 5 |
| replicate_ 3_T0 | 44 | 323 | 14 | 286 | 0 | 34 | 259 | 11 | 290 | 0 |
| replicate_ 3_T12 | 44 |  | 29 |  | 2 | 56 |  | 34 |  | 4 |
| **Experiment 3_ Control_Day 1 (pre-stress)** | | | | | | | | | | |
| replicate_ 1_T0 | 33 | 407 | 12 | 292 | 0 | 33 | 331 | 15 | 305 | 0 |
| replicate_ 1_T12 | 32 |  | 22 |  | 3 | 33 |  | 24 |  | 4 |
| replicate_ 2_T0 | 33 | 233 | 12 | 302 | 0 | 39 | 218 | 16 | 289 | 0 |
| replicate_ 2_T12 | 33 |  | 23 |  | 2 | 37 |  | 24 |  | 2 |
| replicate_ 3_T0 | 35 | 397 | 15 | 325 | 0 | 33 | 345 | 15 | 232 | 0 |
| replicate_ 3_T12 | 34 |  | 23 |  | 3 | 35 |  | 22 |  | 4 |
| **Experiment 1_ Control_Day 4 (stress)** | | | | | | | | | | |
| replicate_ 1_T0 | 51 | 240 | 23 | 292 | 0 | 29 | 225 | 11 | 268 | 0 |
| replicate_ 1_T12 | 30 |  | 23 |  | 4 | 31 |  | 20 |  | 4 |
| replicate_ 2_T0 | 50 | 226 | 17 | 326 | 0 | 35 | 246 | 17 | 301 | 0 |
| replicate_ 2_T12 | 46 |  | 32 |  | 5 | 33 |  | 26 |  | 3 |
| replicate_ 3_T0 | 31 | 336 | 13 | 374 | 0 | 40 | 337 | 14 | 363 | 0 |
| replicate_ 3_T12 | 31 |  | 20 |  | 4 | 32 |  | 17 |  | 4 |
| **Experiment 2_ Control_Day 4 (stress)** | | | | | | | | | | |
| replicate_ 1_T0 | 53 | 251 | 18 | 334 | 0 | 39 | 341 | 13 | 346 | 0 |
| replicate_ 1_T12 | 33 |  | 20 |  | 3 | 36 |  | 23 |  | 2 |
| replicate_ 2_T0 | 44 | 328 | 13 | 339 | 0 | 44 | 296 | 19 | 364 | 0 |
| replicate_ 2_T12 | 50 |  | 34 |  | 3 | 45 |  | 30 |  | 3 |
| replicate_ 3_T0 | 46 | 266 | 21 | 296 | 0 | 44 | 262 | 17 | 224 | 0 |
| replicate_ 3_T12 | 56 |  | 40 |  | 2 | 50 |  | 33 |  | 4 |
| **Experiment 3_ Control_Day 4 (stress)** | | | | | | | | | | |
| replicate_ 1_T0 | 28 | 353 | 12 | 340 | 0 | 36 | 346 | 16 | 350 | 0 |
| replicate_ 1_T12 | 30 |  | 21 |  | 2 | 32 |  | 21 |  | 3 |
| replicate_ 2_T0 | 34 | 293 | 13 | 252 | 0 | 33 | 285 | 13 | 241 | 0 |
| replicate_ 2_T12 | 27 |  | 19 |  | 2 | 34 |  | 23 |  | 3 |
| replicate_ 3_T0 | 33 | 336 | 13 | 241 | 0 | 35 | 322 | 14 | 290 | 0 |
| replicate_ 3_T12 | 37 |  | 26 |  | 4 | 37 |  | 24 |  | 3 |
| **Experiment 1_ Control_Day 8 (recovery)** | | | | | | | | | | |
| replicate_ 1_T0 | 57 | 342 | 19 | 377 | 0 | 66 | 313 | 26 | 420 | 0 |
| replicate_ 1_T12 | 56 |  | 43 |  | 7 | 71 |  | 58 |  | 5 |
| replicate_ 2_T0 | 63 | 336 | 24 | 386 | 0 | 66 | 349 | 25 | 436 | 0 |
| replicate_ 2_T12 | 52 |  | 41 |  | 5 | 54 |  | 38 |  | 3 |
| replicate_ 3_T0 | 58 | 380 | 25 | 442 | 0 | 86 | 399 | 36 | 457 | 0 |
| replicate_ 3_T12 | 67 |  | 52 |  | 5 | 39 |  | 31 |  | 4 |
| **Experiment 2_ Control_Day 8 (recovery)** | | | | | | | | | | |
| replicate_ 1_T0 | 55 | 373 | 20 | 426 | 0 | 60 | 347 | 19 | 399 | 0 |
| replicate_ 1_T12 | 36 |  | 24 |  | 3 | 61 |  | 38 |  | 3 |
| replicate_ 2_T0 | 44 | 413 | 16 | 418 | 0 | 66 | 335 | 25 | 396 | 0 |
| replicate_ 2_T12 | 61 |  | 41 |  | 4 | 44 |  | 26 |  | 4 |
| replicate_ 3_T0 | 50 | 366 | 16 | 445 | 0 | 52 | 316 | 17 | 425 | 0 |
| replicate_ 3_T12 | 49 |  | 31 |  | 4 | 38 |  | 22 |  | 4 |
| **Experiment 3_ Control_Day 8 (recovery)** | | | | | | | | | | |
| replicate_ 1_T0 | 39 | 407 | 16 | 292 | 0 | 35 | 331 | 15 | 305 | 0 |
| replicate_ 1_T12 | 37 |  | 25 |  | 3 | 30 |  | 18 |  | 2 |
| replicate_ 2_T0 | 34 | 451 | 14 | 434 | 0 | 27 | 423 | 10 | 430 | 0 |
| replicate_ 2_T12 | 33 |  | 24 |  | 1 | 35 |  | 26 |  | 4 |
| replicate_ 3_T0 | 32 | 449 | 12 | 405 | 0 | 35 | 455 | 14 | 443 | 0 |
| replicate_ 3_T12 | 47 |  | 31 |  | 2 | 31 |  | 20 |  | 3 |
| **Experiment 1_Temperature_Day 1 (pre-stress)** | | | | | | | | | | |
| replicate_ 1_T0 | 32 | 275 | 11 | 450 | 0 | 26 | 190 | 15 | 452 | 0 |
| replicate_ 1_T12 | 32 |  | 22 |  | 6 | 28 |  | 22 |  | 7 |
| replicate_ 2_T0 | 39 | 258 | 18 | 396 | 0 | 43 | 305 | 19 | 400 | 0 |
| replicate_ 2_T12 | 35 |  | 30 |  | 4 | 30 |  | 26 |  | 12 |
| replicate_ 3_T0 | 30 | 269 | 11 | 832 | 0 | 19 | 132 | 5 | 865 | 0 |
| replicate_ 3_T12 | 50 |  | 44 |  | 17 | 47 |  | 37 |  | 3 |
| **Experiment 2_ Temperature _Day 1 (pre-stress)** | | | | | | | | | | |
| replicate_ 1_T0 | 52 | 272 | 20 | 126 | 0 | 47 | 256 | 18 | 144 | 0 |
| replicate_ 1_T12 | 28 |  | 20 |  | 3 | 33 |  | 23 |  | 2 |
| replicate_ 2_T0 | 43 | 350 | 15 | 227 | 0 | 26 | 333 | 9 | 233 | 0 |
| replicate_ 2_T12 | 37 |  | 26 |  | 3 | 33 |  | 22 |  | 1 |
| replicate_ 3_T0 | 52 | 251 | 22 | 202 | 0 | 34 | 267 | 15 | 204 | 0 |
| replicate_ 3_T12 | 36 |  | 25 |  | 1 | 38 |  | 27 |  | 3 |
| **Experiment 3_ Temperature _Day 1 (pre-stress)** | | | | | | | | | | |
| replicate_ 1_T0 | 37 | 317 | 15 | 291 | 0 | 32 | 315 | 14 | 329 | 0 |
| replicate_ 1_T12 | 28 |  | 20 |  | 3 | 30 |  | 21 |  | 2 |
| replicate_ 2_T0 | 34 | 288 | 14 | 335 | 0 | 44 | 315 | 19 | 326 | 0 |
| replicate_ 2_T12 | 33 |  | 23 |  | 2 | 35 |  | 25 |  | 4 |
| replicate_ 3_T0 | 37 | 356 | 15 | 357 | 0 | 32 | 335 | 12 | 349 | 0 |
| replicate_ 3_T12 | 36 |  | 25 |  | 4 | 32 |  | 21 |  | 4 |
| **Experiment 1_Temperature l_Day 4 (stress)** | | | | | | | | | | |
| replicate_ 1_T0 | 24 | 140 | 12 | 460 | 0 | 77 | 182 | 22 | 473 | 0 |
| replicate_ 1_T12 | 21 |  | 16 |  | 5 | 36 |  | 23 |  | 5 |
| replicate_ 2_T0 | 29 | 117 | 11 | 447 | 0 | 26 | 122 | 7 | 448 | 0 |
| replicate_ 2_T12 | 37 |  | 25 |  | 6 | 28 |  | 20 |  | 3 |
| replicate_ 3_T0 | 24 | 293 | 5 | 371 | 0 | 34 | 271 | 11 | 371 | 0 |
| replicate_ 3_T12 | 29 |  | 19 |  | 3 | 36 |  | 24 |  | 3 |
| **Experiment 2_Temperature _Day 4 (stress)** | | | | | | | | | | |
| replicate_ 1_T0 | 47 | 384 | 19 | 411 | 0 | 39 | 329 | 13 | 393 | 0 |
| replicate_ 1_T12 | 41 |  | 30 |  | 3 | 40 |  | 24 |  | 2 |
| replicate_ 2_T0 | 51 | 314 | 19 | 272 | 0 | 44 | 346 | 20 | 343 | 0 |
| replicate_ 2_T12 | 54 |  | 35 |  | 4 | 52 |  | 38 |  | 3 |
| replicate_ 3_T0 | 42 | 256 | 18 | 431 | 0 | 46 | 244 | 18 | 449 | 0 |
| replicate_ 3_T12 | 61 |  | 40 |  | 4 | 50 |  | 37 |  | 1 |
| **Experiment 3_Temperature _Day 4 (stress)** | | | | | | | | | | |
| replicate_ 1_T0 | 28 | 351 | 12 | 298 | 0 | 33 | 331 | 15 | 297 | 0 |
| replicate_ 1_T12 | 33 |  | 24 |  | 1 | 33 |  | 23 |  | 2 |
| replicate_ 2_T0 | 25 | 341 | 10 | 364 | 0 | 35 | 346 | 14 | 364 | 0 |
| replicate_ 2_T12 | 33 |  | 22 |  | 3 | 33 |  | 23 |  | 3 |
| replicate_ 3_T0 | 33 | 325 | 13 | 312 | 0 | 33 | 326 | 14 | 312 | 0 |
| replicate_ 3_T12 | 35 |  | 21 |  | 3 | 38 |  | 24 |  | 2 |
| **Experiment 1_Temperature _Day 8 (recovery)** | | | | | | | | | | |
| replicate_ 1_T0 | 43 | 447 | 13 | 468 | 0 | 29 | 425 | 9 | 395 | 0 |
| replicate_ 1_T12 | 41 |  | 28 |  | 1 | 31 |  | 22 |  | 4 |
| replicate_ 2_T0 | 67 | 304 | 25 | 384 | 0 | 49 | 289 | 22 | 298 | 0 |
| replicate_ 2_T12 | 52 |  | 35 |  | 3 | 35 |  | 22 |  | 2 |
| replicate_ 3_T0 | 40 | 309 | 15 | 362 | 0 | 34 | 318 | 13 | 366 | 0 |
| replicate_ 3_T12 | 45 |  | 34 |  | 4 | 22 |  | 17 |  | 3 |
| **Experiment 2_ Temperature _Day 8 (recovery)** | | | | | | | | | | |
| replicate_ 1_T0 | 51 | 248 | 18 | 442 | 0 | 53 | 267 | 19 | 442 | 0 |
| replicate_ 1_T12 | 67 |  | 46 |  | 8 | 61 |  | 45 |  | 5 |
| replicate_ 2_T0 | 55 | 417 | 25 | 521 | 0 | 55 | 391 | 19 | 516 | 0 |
| replicate_ 2_T12 | 52 |  | 38 |  | 5 | 54 |  | 34 |  | 5 |
| replicate_ 3_T0 | 61 | 362 | 25 | 228 | 0 | 40 | 576 | 12 | 560 | 0 |
| replicate_ 3_T12 | 54 |  | 38 |  | 4 | 53 |  | 26 |  | 5 |
| **Experiment 3_Temperature _Day 8 (recovery)** | | | | | | | | | | |
| replicate_ 1_T0 | 40 | 373 | 17 | 383 | 0 | 39 | 374 | 16 | 380 | 0 |
| replicate_ 1_T12 | 38 |  | 25 |  | 4 | 42 |  | 27 |  | 4 |
| replicate_ 2_T0 | 36 | 557 | 13 | 579 | 0 | 29 | 555 | 9 | 549 | 0 |
| replicate_ 2_T12 | 34 |  | 24 |  | 3 | 33 |  | 21 |  | 3 |
| replicate_ 3_T0 | 31 | 400 | 11 | 383 | 0 | 35 | 436 | 12 | 384 | 0 |
| replicate_ 3_T12 | 40 |  | 26 |  | 4 | 46 |  | 28 |  | 2 |
| **Experiment 1_Salt_Day 1 (pre-stress)** | | | | | | | | | | |
| replicate_ 1_T0 | 19 | 315 | 8 | 232 | 0 | 52 | 328 | 28 | 261 | 0 |
| replicate_ 1_T12 | 45 |  | 34 |  | 3 | 74 |  | 63 |  | 9 |
| replicate_ 2_T0 | 30 | 98 | 7 | 271 | 2 | 36 | 119 | 11 | 292 | 0 |
| replicate_ 2_T12 | 32 |  | 27 |  | 14 | 44 |  | 38 |  | 11 |
| replicate_ 3_T0 | 52 | 264 | 19 | 285 | 0 | 33 | 299 | 11 | 291 | 0 |
| replicate_ 3_T12 | 30 |  | 23 |  | 4 | 48 |  | 37 |  | 10 |
| **Experiment 2_ Salt _Day 1 (pre-stress)** | | | | | | | | | | |
| replicate_ 1_T0 | 39 | 299 | 15 | 271 | 0 | 40 | 295 | 15 | 242 | 0 |
| replicate_ 1_T12 | 43 |  | 29 |  | 3 | 53 |  | 36 |  | 2 |
| replicate_ 2_T0 | 38 | 230 | 16 | 288 | 0 | 40 | 228 | 16 | 318 | 0 |
| replicate_ 2_T12 | 31 |  | 21 |  | 3 | 35 |  | 23 |  | 3 |
| replicate_ 3_T0 | 41 | 224 | 14 | 311 | 0 | 39 | 228 | 16 | 286 | 0 |
| replicate_ 3_T12 | 41 |  | 26 |  | 3 | 36 |  | 25 |  | 4 |
| **Experiment 3_Salt_Day 1 (pre-stress)** | | | | | | | | | | |
| replicate_ 1_T0 | 33 | 400 | 11 | 341 | 0 | 39 | 412 | 13 | 294 | 0 |
| replicate_ 1_T12 | 34 |  | 24 |  | 3 | 41 |  | 28 |  | 3 |
| replicate_ 2_T0 | 36 | 391 | 15 | 361 | 0 | 35 | 395 | 15 | 260 | 0 |
| replicate_ 2_T12 | 42 |  | 26 |  | 3 | 32 |  | 19 |  | 2 |
| replicate_ 3_T0 | 30 | 333 | 13 | 290 | 0 | 35 | 345 | 14 | 297 | 0 |
| replicate_ 3_T12 | 37 |  | 25 |  | 3 | 42 |  | 27 |  | 3 |
| **Experiment 1_Salt_Day 4 (stress)** | | | | | | | | | | |
| replicate_ 1_T0 | 51 | 378 | 21 | 302 | 0 | 72 | 446 | 31 | 371 | 0 |
| replicate_ 1_T12 | 37 |  | 23 |  | 2 | 28 |  | 12 |  | 2 |
| replicate_ 2_T0 | 42 | 351 | 16 | 319 | 0 | 33 | 318 | 9 | 329 | 0 |
| replicate_ 2_T12 | 59 |  | 38 |  | 4 | 33 |  | 9 |  | 2 |
| replicate_ 3_T0 | 38 | 365 | 15 | 357 | 0 | 37 | 321 | 12 | 318 | 0 |
| replicate_ 3_T12 | 28 |  | 21 |  | 3 | 40 |  | 15 |  | 3 |
| **Experiment 2_Salt _Day 4 (stress)** | | | | | | | | | | |
| replicate_ 1_T0 | 37 | 278 | 13 | 352 | 0 | 37 | 329 | 13 | 352 | 0 |
| replicate_ 1_T12 | 34 |  | 22 |  | 4 | 33 |  | 12 |  | 6 |
| replicate_ 2_T0 | 34 | 267 | 11 | 315 | 0 | 34 | 248 | 13 | 301 | 0 |
| replicate_ 2_T12 | 36 |  | 22 |  | 4 | 34 |  | 13 |  | 6 |
| replicate_ 3_T0 | 41 | 255 | 15 | 356 | 0 | 36 | 263 | 13 | 337 | 0 |
| replicate_ 3_T12 | 37 |  | 24 |  | 4 | 40 |  | 15 |  | 6 |
| **Experiment 3_ Salt _Day 4 (stress)** | | | | | | | | | | |
| replicate_ 1_T0 | 43 | 366 | 16 | 353 | 0 | 37 | 364 | 13 | 363 | 0 |
| replicate_ 1_T12 | 42 |  | 29 |  | 4 | 39 |  | 14 |  | 6 |
| replicate_ 2_T0 | 47 | 374 | 20 | 345 | 0 | 40 | 383 | 15 | 362 | 0 |
| replicate_ 2_T12 | 40 |  | 27 |  | 3 | 46 |  | 18 |  | 5 |
| replicate_ 3_T0 | 33 | 352 | 14 | 368 | 0 | 44 | 359 | 16 | 377 | 0 |
| replicate_ 3_T12 | 35 |  | 25 |  | 3 | 42 |  | 15 |  | 6 |
| **Experiment 1_ Salt _Day 8 (recovery)** | | | | | | | | | | |
| replicate_ 1_T0 | 28 | 490 | 9 | 493 | 0 | 49 | 456 | 16 | 506 | 0 |
| replicate_ 1_T12 | 47 |  | 35 |  | 1 | 44 |  | 32 |  | 2 |
| replicate_ 2_T0 | 51 | 413 | 20 | 415 | 0 | 41 | 377 | 17 | 398 | 0 |
| replicate_ 2_T12 | 47 |  | 32 |  | 3 | 62 |  | 40 |  | 5 |
| replicate_ 3_T0 | 48 | 388 | 20 | 461 | 0 | 58 | 427 | 25 | 519 | 0 |
| replicate_ 3_T12 | 43 |  | 35 |  | 2 | 43 |  | 33 |  | 3 |
| **Experiment 2_ Salt _Day 8 (recovery)** | | | | | | | | | | |
| replicate_ 1_T0 | 42 | 250 | 14 | 418 | 0 | 79 | 243 | 28 | 369 | 0 |
| replicate_ 1_T12 | 73 |  | 46 |  | 7 | 67 |  | 44 |  | 9 |
| replicate_ 2_T0 | 53 | 321 | 18 | 421 | 0 | 76 | 347 | 26 | 285 | 0 |
| replicate_ 2_T12 | 75 |  | 46 |  | 8 | 66 |  | 45 |  | 12 |
| replicate_ 3_T0 | 62 | 290 | 25 | 332 | 0 | 76 | 379 | 33 | 344 | 0 |
| replicate_ 3_T12 | 53 |  | 37 |  | 4 | 87 |  | 51 |  | 13 |
| **Experiment 3_ Salt _Day 8 (recovery)** | | | | | | | | | | |
| replicate_ 1_T0 | 27 | 379 | 11 | 360 | 0 | 30 | 350 | 11 | 371 | 0 |
| replicate_ 1_T12 | 28 |  | 20 |  | 3 | 34 |  | 22 |  | 3 |
| replicate_ 2_T0 | 44 | 393 | 18 | 427 | 0 | 55 | 397 | 23 | 393 | 0 |
| replicate_ 2_T12 | 39 |  | 27 |  | 3 | 30 |  | 21 |  | 3 |
| replicate_ 3_T0 | 34 | 381 | 13 | 385 | 0 | 31 | 382 | 12 | 389 | 0 |
| replicate_ 3_T12 | 30 |  | 19 |  | 2 | 38 |  | 24 |  | 3 |
| **Experiment 1_Combination_Day 1 (pre-stress)** | | | | | | | | | | |
| replicate_ 1_T0 | 63 | 246 | 19 | 429 | 0 | 44 | 141 | 16 | 391 | 0 |
| replicate_ 1_T12 | 41 |  | 31 |  | 4 | 56 |  | 38 |  | 14 |
| replicate_ 2_T0 | 35 | 112 | 16 | 294 | 0 | 44 | 522 | 19 | 362 | 0 |
| replicate_ 2_T12 | 38 |  | 32 |  | 6 | 45 |  | 37 |  | 17 |
| replicate_ 3_T0 | 45 | 201 | 22 | 283 | 0 | 37 | 163 | 15 | 263 | 0 |
| replicate_ 3_T12 | 45 |  | 31 |  | 10 | 34 |  | 26 |  | 8 |
| **Experiment 2_ Combination _Day 1 (pre-stress)** | | | | | | | | | | |
| replicate_ 1_T0 | 62 | 345 | 24 | 279 | 0 | 48 | 387 | 19 | 299 | 0 |
| replicate_ 1_T12 | 33 |  | 25 |  | 2 | 40 |  | 30 |  | 3 |
| replicate_ 2_T0 | 35 | 329 | 12 | 270 | 0 | 43 | 295 | 16 | 350 | 0 |
| replicate_ 2_T12 | 35 |  | 24 |  | 2 | 33 |  | 23 |  | 2 |
| replicate_ 3_T0 | 56 | 395 | 19 | 388 | 0 | 41 | 371 | 15 | 336 | 0 |
| replicate_ 3_T12 | 37 |  | 26 |  | 2 | 33 |  | 22 |  | 4 |
| **Experiment 3_ Combination _Day 1 (pre-stress)** | | | | | | | | | | |
| replicate_ 1_T0 | 44 | 374 | 18 | 383 | 0 | 37 | 380 | 15 | 401 | 0 |
| replicate_ 1_T12 | 40 |  | 27 |  | 4 | 34 |  | 23 |  | 3 |
| replicate_ 2_T0 | 42 | 381 | 18 | 253 | 0 | 37 | 390 | 17 | 362 | 0 |
| replicate_ 2_T12 | 32 |  | 22 |  | 3 | 37 |  | 27 |  | 4 |
| replicate_ 3_T0 | 44 | 380 | 16 | 364 | 0 | 38 | 372 | 15 | 375 | 0 |
| replicate_ 3_T12 | 46 |  | 31 |  | 3 | 38 |  | 25 |  | 3 |
| **Experiment 1_ Combination _Day 4 (stress)** | | | | | | | | | | |
| replicate_ 1_T0 | 36 | 259 | 12 | 407 | 0 | 41 | 244 | 12 | 375 | 0 |
| replicate_ 1_T12 | 57 |  | 38 |  | 10 | 34 |  | 12 |  | 9 |
| replicate_ 2_T0 | 50 | 141 | 16 | 460 | 0 | 35 | 155 | 10 | 434 | 0 |
| replicate_ 2_T12 | 31 |  | 22 |  | 2 | 37 |  | 12 |  | 3 |
| replicate_ 3_T0 | 35 | 258 | 13 | 350 | 0 | 37 | 280 | 9 | 343 | 0 |
| replicate_ 3_T12 | 29 |  | 18 |  | 2 | 37 |  | 11 |  | 1 |
| **Experiment 2_ Combination _Day 4 (stress)** | | | | | | | | | | |
| replicate_ 1_T0 | 38 | 330 | 14 | 320 | 0 | 36 | 329 | 13 | 318 | 0 |
| replicate_ 1_T12 | 34 |  | 22 |  | 4 | 40 |  | 15 |  | 3 |
| replicate_ 2_T0 | 42 | 272 | 13 | 296 | 0 | 47 | 285 | 15 | 317 | 0 |
| replicate_ 2_T12 | 38 |  | 24 |  | 4 | 42 |  | 15 |  | 4 |
| replicate_ 3_T0 | 34 | 306 | 13 | 349 | 0 | 48 | 305 | 18 | 331 | 0 |
| replicate_ 3_T12 | 38 |  | 24 |  | 3 | 46 |  | 19 |  | 5 |
| **Experiment 3_ Combination _Day 4 (stress)** | | | | | | | | | | |
| replicate_ 1_T0 | 35 | 383 | 14 | 372 | 0 | 37 | 363 | 14 | 330 | 0 |
| replicate_ 1_T12 | 35 |  | 25 |  | 2 | 35 |  | 14 |  | 5 |
| replicate_ 2_T0 | 41 | 356 | 16 | 282 | 0 | 37 | 355 | 15 | 290 | 0 |
| replicate_ 2_T12 | 38 |  | 23 |  | 2 | 30 |  | 13 |  | 2 |
| replicate_ 3_T0 | 40 | 355 | 16 | 322 | 0 | 38 | 365 | 15 | 305 | 0 |
| replicate_ 3_T12 | 29 |  | 19 |  | 2 | 40 |  | 17 |  | 4 |
| **Experiment 1_ Combination _Day 8 (recovery)** | | | | | | | | | | |
| replicate_ 1_T0 | 75 | 285 | 27 | 333 | 0 | 87 | 249 | 34 | 325 | 0 |
| replicate_ 1_T12 | 80 |  | 55 |  | 6 | 87 |  | 64 |  | 11 |
| replicate_ 2_T0 | 57 | 354 | 26 | 387 | 0 | 52 | 326 | 23 | 460 | 0 |
| replicate_ 2_T12 | 71 |  | 52 |  | 6 | 38 |  | 25 |  | 4 |
| replicate_ 3_T0 | 47 | 369 | 18 | 461 | 0 | 50 | 278 | 14 | 480 | 0 |
| replicate_ 3_T12 | 45 |  | 34 |  | 5 | 59 |  | 35 |  | 4 |
| **Experiment 2_ Combination _Day 8 (recovery)** | | | | | | | | | | |
| replicate_ 1_T0 | 63 | 371 | 22 | 431 | 0 | 63 | 379 | 26 | 452 | 0 |
| replicate_ 1_T12 | 57 |  | 38 |  | 5 | 70 |  | 44 |  | 8 |
| replicate_ 2_T0 | 66 | 388 | 26 | 465 | 0 | 72 | 387 | 25 | 491 | 0 |
| replicate_ 2_T12 | 40 |  | 25 |  | 4 | 38 |  | 23 |  | 4 |
| replicate_ 3_T0 | 38 | 381 | 16 | 505 | 0 | 47 | 403 | 19 | 474 | 0 |
| replicate_ 3_T12 | 55 |  | 40 |  | 5 | 51 |  | 35 |  | 4 |
| **Experiment 3_ Combination _Day 8 (recovery)** | | | | | | | | | | |
| replicate_ 1_T0 | 36 | 347 | 12 | 348 | 0 | 27 | 332 | 10 | 348 | 0 |
| replicate_ 1_T12 | 33 |  | 20 |  | 2 | 29 |  | 19 |  | 3 |
| replicate_ 2_T0 | 42 | 339 | 17 | 343 | 0 | 41 | 342 | 16 | 348 | 0 |
| replicate_ 2_T12 | 32 |  | 20 |  | 3 | 31 |  | 18 |  | 2 |
| replicate_ 3_T0 | 39 | 354 | 15 | 361 | 0 | 33 | 366 | 12 | 367 | 0 |
| replicate_ 3_T12 | 43 |  | 29 |  | 3 | 47 |  | 30 |  | 3 |

**Table S5.** Summary of read and OTU Counts at different processing stages for 16S and 18S data

| **processing stage** | **16S data** | **18S data** |
| --- | --- | --- |
| after processing (Natrix2 pipeline) | 141,360,383 reads /  74,747 OTUs | 85,637,016 reads /  9,435 OTUs |
| after removal of negative controls | 78,637,803 reads /  35,480 OTUs | 65,192,803 reads /  4,193 OTUs |
| after noise filtering | 74,108,729 reads /  7,929 OTUs | 62,520,360 reads /  2,038 OTUs |


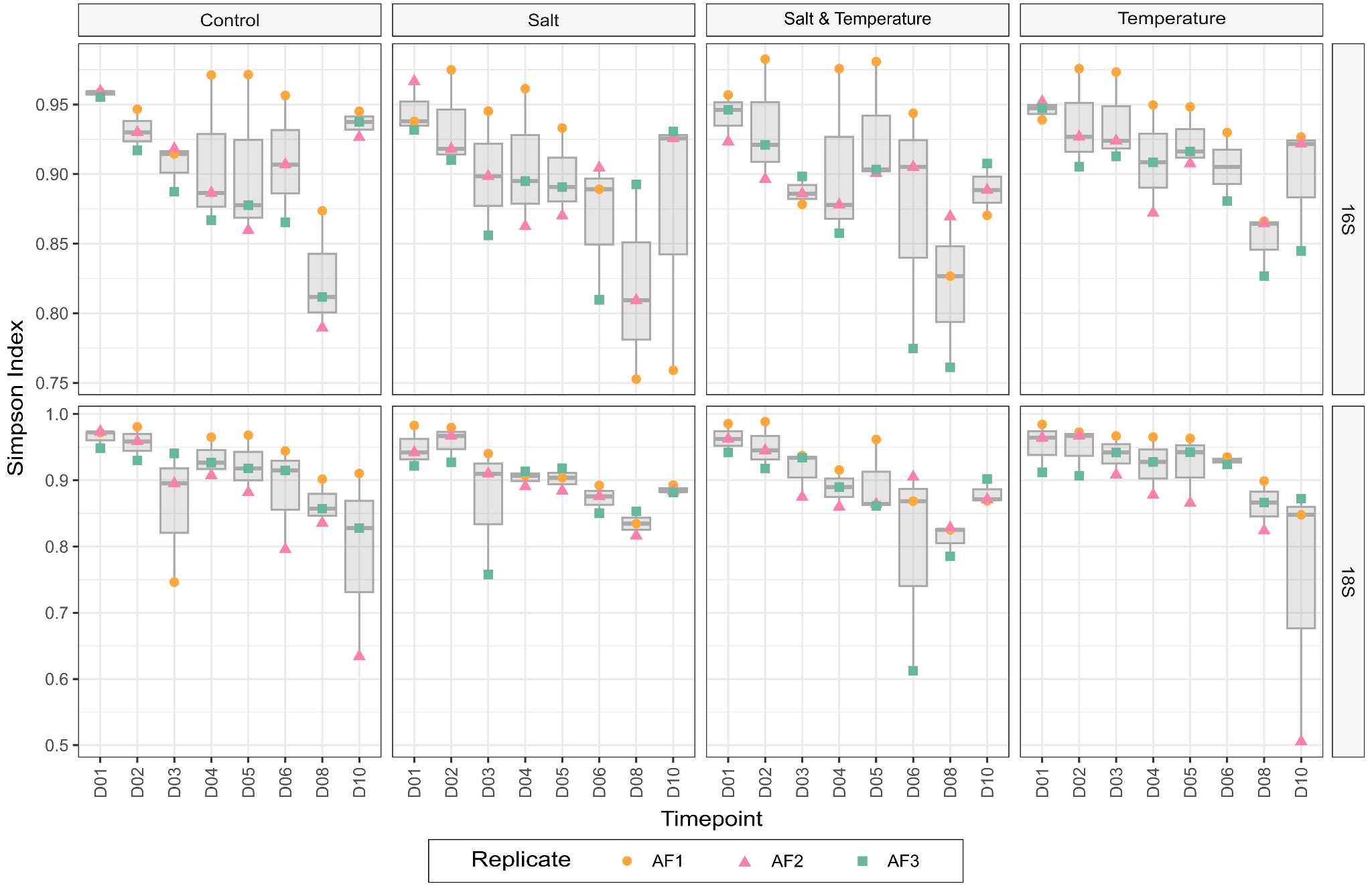


**Figure S2.** Simpson’s Diversity Index for the prokaryotic (top) and eukaryotic microbial (bottom) communities in the different treatments during the experiments.


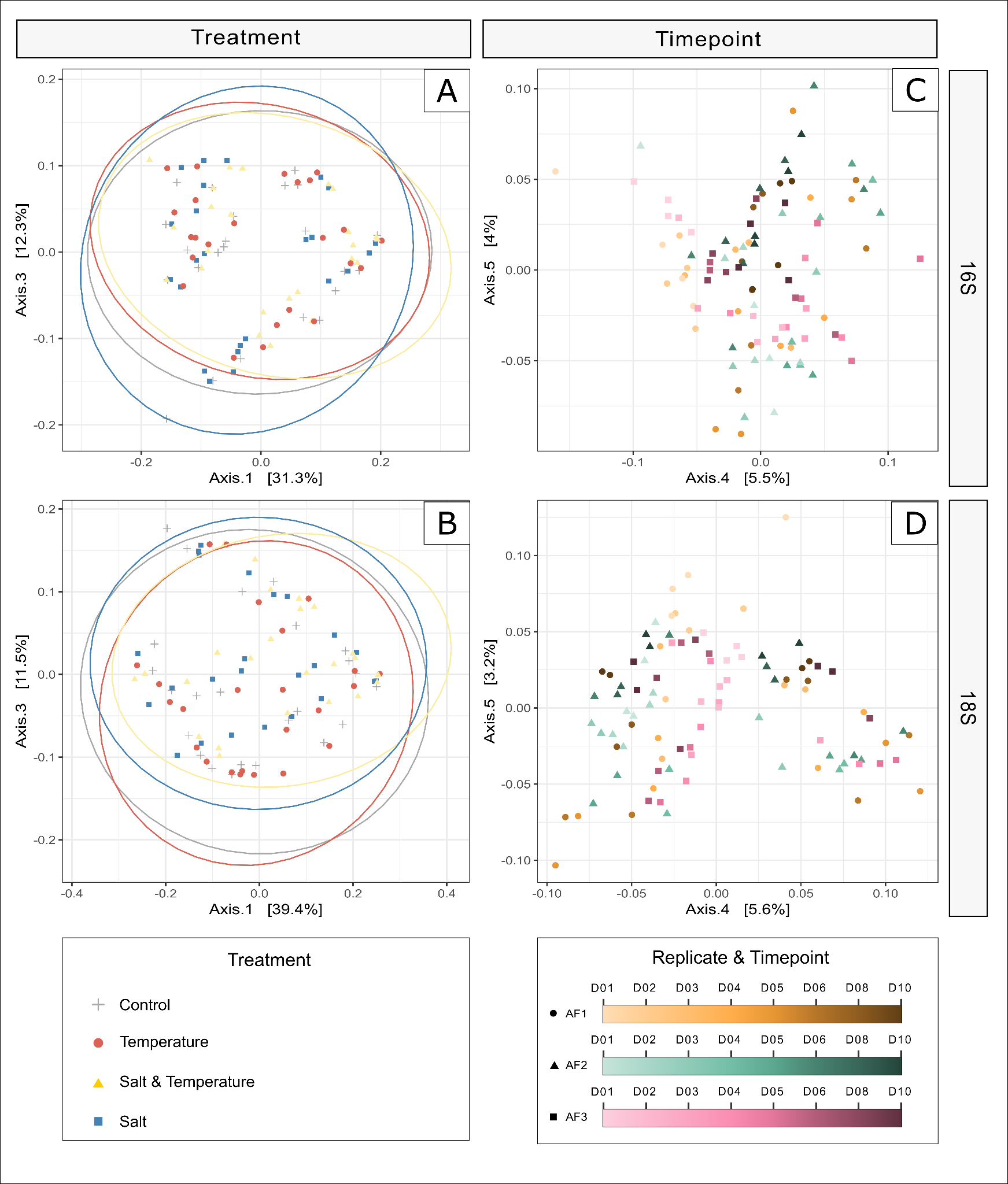


**Figure S3.** Principal coordinates analysis (PCoA) of community composition based on Bray-Curtis dissimilarity measures in water samples from all three replicates. The PCoA plots depict the community composition in the different treatments on axes 1 and 3 for the prokaryotic (16S; A) and the microbial eukaryotic community (18S; B) as well as stressor samples from the replicates on the axes 4 and 5 for the prokaryotic (16S; C) and the eukaryotic microbial (18S; D) communities.


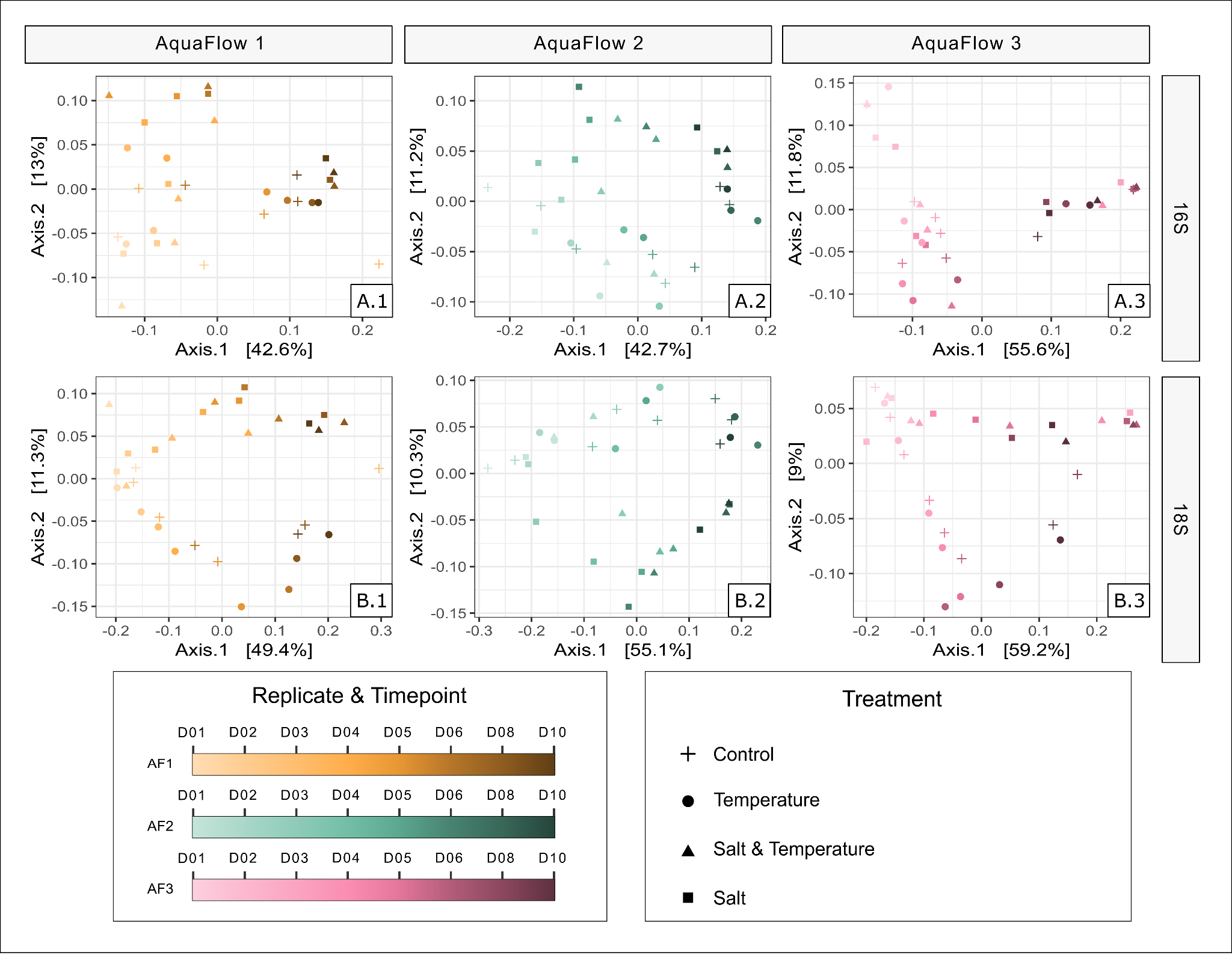


**Figure S4.** Principal coordinates analysis (PCoA) of community composition in water samples based on Bray-Curtis dissimilarity measures from the individual replicates. The PCoA plots depict temporal effect on axis 1 and the variation among the replicates in the community composition of the prokaryotic (16S; A.1-3) and eukaryotic microbial (18S; B.1-3) community.


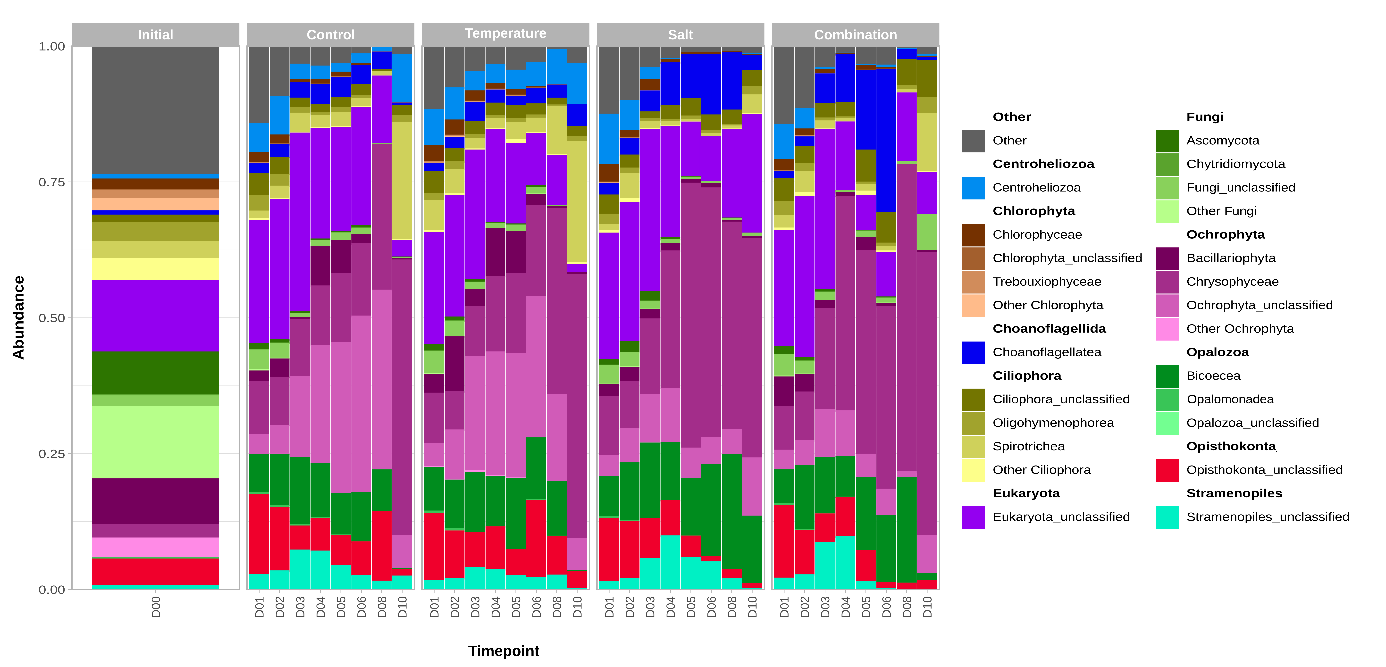
**Figure S5.** Taxonomic composition of the microeukaryotic community. Bar plots display the ten most abundant phyla and their three most abundant classes. Classes within the same phylum are represented by similar colors. 'Other' encompasses all additional taxa present in the samples. Samples were collected on the first day of the 10-day acclimation period (D00), after acclimation but before stressor addition (D01), during the stressor phase (D02-D05), and after stressor removal (D06-D10).


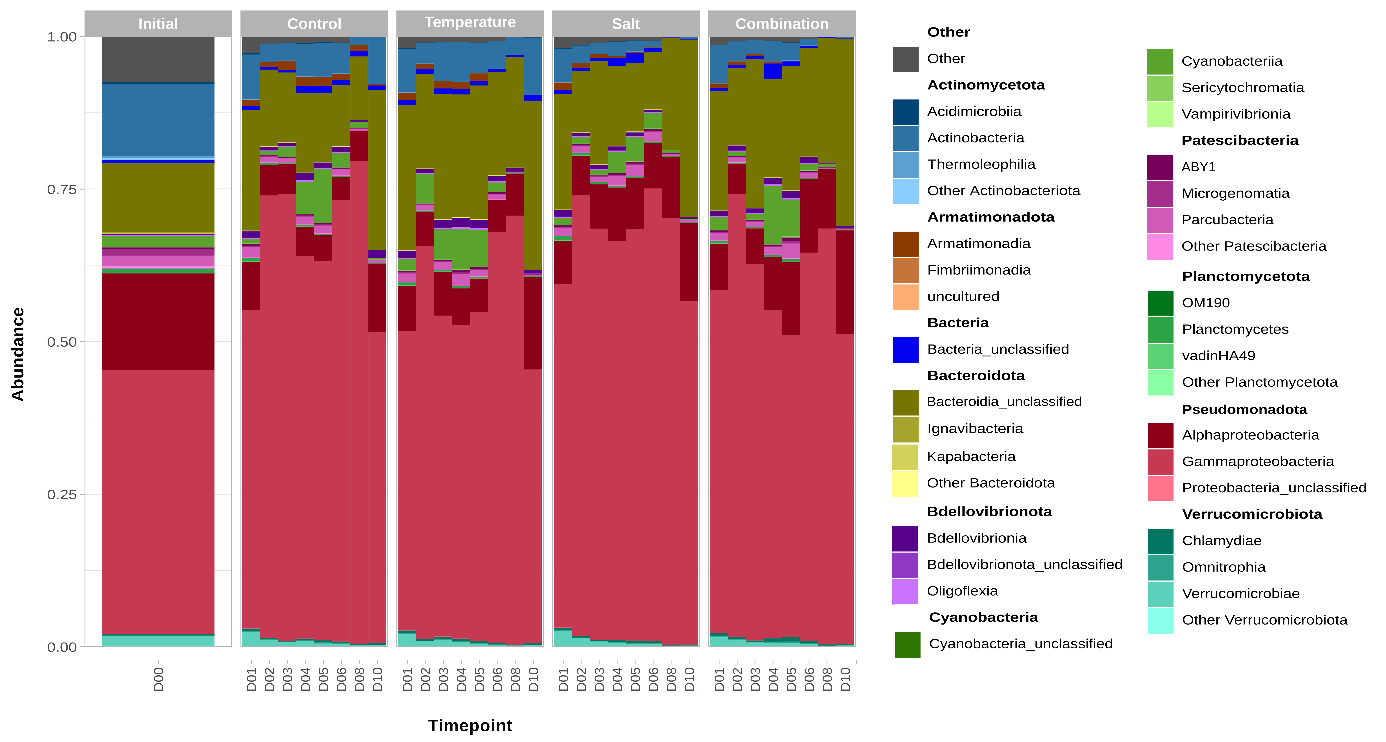


**Figure S6.** Taxonomic composition of the prokaryotic community. Bar plots display the ten most abundant phyla and their three most abundant classes. Classes within the same phylum are represented by similar colors. 'Other' encompasses all additional taxa present in the samples. Samples were collected on the first day of the 10-day acclimation period (D00), after acclimation but before stressor addition (D01), during the stressor phase (D02-D05), and after stressor removal (D06-D10).
